# Diagnostic Accuracy of Artificial Intelligence-Based Electrocardiography for the Detection of Heart Diseases: A Systematic Review and Meta-Analysis

**DOI:** 10.3390/diagnostics16183014

**Published:** 2026-09-17

**Authors:** Joshuan J. Barboza, Oscar Andres Ramirez-Teran, Eduardo Tomás-Alvarado, Carlos A. Barba, Julián Santa Cruz-Venegas, Carmen Ayala-Jara, Maicol Cortez-Sandoval, Bryam López Tuesta, Euler Tito Chura, Oscar Alexander Braulio Hernández Rios, Oriana Rivera-Lozada, Cesar Bonilla-Asalde

**Affiliations:** 1 Vicerrectorado de Investigación, Universidad Señor de Sipán, Chiclayo 14001, Peru; riveraoriana@uss.edu.pe (O.R.-L.);; 2 Hospital de Pediatría “Dr. Silvestre Frenk Freund”, Instituto Mexicano del Seguro Social, Ciudad de México 06720, Mexico; 3 Hospital General Regional 17, Instituto Mexicano del Seguro Social, Cancún 77533, Mexico; 4 Hospital d’Olot i Comarcal de la Garrotxa, 17800 Olot, Spain; 5 Facultad de medicina, Universidad Tecnológica de las Islas Canarias, 38108 San Cristóbal de La Laguna, Spain; julian.adan35@gmail.com; 6 Facultad de Farmacia y Bioquímica, Universidad Nacional de Trujillo, Trujillo 13011, Peru; 7 Escuela de Medicina Humana, Universidad Científica del Sur, Lima 15067, Peru; 8 Departamento de Cardiología, Hospital Nacional Edgardo Rebagliati, Lima 15072, Peru; 9 Departamento de Ingeniería de Sistemas e Informática, Universidad Nacional de Moquegua, Moquegua 18611, Peru; 10 Facultad de medicina, Universidad Ricardo Palma, Lima 15039, Peru

**Keywords:** artificial intelligence, deep learning, electrocardiography, heart failure, diagnostic test accuracy, systematic review, meta-analysis

## Abstract

**Background/Objectives**: Electrocardiography augmented by artificial intelligence (AI-ECG) has been put forward as an inexpensive, scalable means of identifying a wide range of cardiac disorders, but reported performance differs markedly with the condition targeted, the algorithm, and the reference standard. Our pre-specified primary objective was to estimate the diagnostic accuracy of AI-ECG for heart failure/left ventricular systolic dysfunction (HF/LVSD); accuracy in other cardiac conditions was examined condition by condition, and a cross-condition estimate was computed only as a secondary, descriptive summary. **Methods**: Following the PRISMA-DTA statement, we systematically reviewed diagnostic test accuracy (DTA) studies indexed in PubMed, Scopus, Web of Science, and Embase from inception to 31 March 2026. Eligible records were primary cohort or case–control diagnostic studies applying an AI algorithm to the ECG against an acceptable reference standard, with a reconstructable 2 × 2 table. Screening, extraction, and QUADAS-2 appraisal were performed independently in duplicate. Sensitivity and specificity were pooled with a bivariate random-effects (Reitsma) model whenever four or more studies shared a target condition, with the corresponding hierarchical summary receiver operating characteristic (HSROC) curve; subgroups with fewer than four studies were synthesised descriptively. Pre-specified sensitivity analyses excluded studies at high risk of bias. Certainty was graded with GRADE for test accuracy. **Results**: Twenty studies (155,442 ECG–reference pairs) were included and 12 (125,568 ECG–reference pairs) entered the quantitative synthesis. In the pre-specified HF/LVSD analysis (k = 5; n = 101,875), pooled sensitivity was 0.86 (95% confidence interval [CI], 0.80–0.90) and pooled specificity was 0.79 (95% CI, 0.70–0.85), with an HSROC area under the curve (AUC) of 0.89; restricting the analysis to the three studies at low risk of bias gave a sensitivity of 0.83 (95% CI, 0.81–0.85) and a specificity of 0.82 (95% CI, 0.75–0.87). The estimated between-study correlation between logit-sensitivity and logit-specificity was −0.86, indicating a strong implicit threshold effect that the bivariate/HSROC framework accommodates. Descriptive subgroup results indicated high accuracy for atrial fibrillation (median sensitivity 0.96, median specificity 0.94; k = 2) and inconsistent performance across coronary disease and acute coronary syndromes (median sensitivity 0.80, median specificity 0.85; k = 2). The secondary cross-condition estimate (k = 12) was 0.84 (95% CI, 0.77–0.89) for sensitivity and 0.87 (95% CI, 0.79–0.93) for specificity (HSROC-AUC 0.92); because it spans heterogeneous targets it is reported as a descriptive summary only. Overall risk of bias was low in 3/20 studies, unclear in 4/20, and high in 13/20, chiefly in the index test and reference standard domains. The Deeks test indicated funnel asymmetry (*p* = 0.022), which in DTA reviews commonly reflects heterogeneity in true accuracy rather than publication bias. **Conclusions**: For HF/LVSD, AI-ECG shows consistent and reproducible accuracy against echocardiography, and this estimate proved robust to the exclusion of studies at high risk of bias. Evidence for other cardiac conditions remains condition specific and less certain. Wider adoption would benefit from prospective external validation against guideline-based reference standards, independent adjudication, and transparent reporting of 2 × 2 data at pre-specified operating points.

## 1. Introduction

Worldwide, cardiovascular diseases continue to be the foremost source of illness and death, causing roughly 18 million deaths each year and constituting the largest single driver of global disability-adjusted life-years [1,2]. Heart failure (HF), arrhythmias such as atrial fibrillation, and acute coronary syndromes together account for an outsized proportion of hospital admissions and avoidable mortality within this burden [2,3]. Because it is cheap, broadly accessible, and reproducible, the 12-lead electrocardiogram (ECG) has long been the central diagnostic investigation in cardiovascular medicine. Standard visual reading, however, is hampered by variability between observers and by the human eye’s inability to perceive the subtle morphological cues that can foreshadow clinically apparent disease [4].

Artificial intelligence (AI)—especially deep learning applied directly to the raw ECG waveform or image—has emerged as a means of detecting patterns that lie beyond conventional electrocardiographic criteria [4,5]. In an influential report, Attia and colleagues trained a convolutional neural network that identified asymptomatic left ventricular systolic dysfunction (LVSD) from the routine 12-lead ECG, achieving an area under the receiver operating characteristic curve (AUC) close to 0.93 [6], and later randomised trials indicated that embedding such algorithms in clinical workflows can speed up the recognition of low ejection fraction [7]. Analogous AI methods have since been built for atrial fibrillation [8], hypertrophic cardiomyopathy [9], left ventricular hypertrophy, coronary artery disease, and acute myocardial infarction [4,5].

The field has been surveyed repeatedly over the past two years. Narrative and scoping reviews have traced the trajectory of AI-ECG from methodological proof-of-concept to prospective deployment and have catalogued its emerging roles in opportunistic screening, risk stratification, and workflow acceleration [10]; parallel reviews have mapped AI applications across atherosclerotic cardiovascular disease [11] and interventional cardiology [12]. These syntheses are informative about scope and about the translational agenda, but they are not quantitative accuracy reviews: they do not extract 2 × 2 contingency data, do not appraise studies with QUADAS-2, and therefore cannot separate discrimination measured on a development cohort from accuracy at a deployable operating point. Where pooled DTA estimates do exist, they are typically confined to a single target—atrial fibrillation above all—and the accuracy attributed to AI-ECG differs markedly with the condition under study, the architecture of the algorithm, the ECG modality (single- versus multi-lead), the training data, and the reference standard chosen to define truth [4,5].

To address this gap, we performed a systematic review and meta-analysis of diagnostic test accuracy with four aims: (i) to derive a pre-specified pooled estimate for HF/LVSD, which we designated in advance as the primary clinical question; (ii) to synthesise, condition by condition, the diagnostic accuracy of AI-based ECG for other cardiac targets; (iii) to investigate heterogeneity by condition, ECG modality, AI architecture, decision threshold, and clinical setting; and (iv) to evaluate risk of bias and the certainty of the evidence.

## 2. Materials and Methods

### 2.1. Protocol and Registration

Both the conduct and the reporting of this review followed the Preferred Reporting Items for Systematic Reviews and Meta-Analyses of Diagnostic Test Accuracy Studies (PRISMA-DTA) [13] together with the PRISMA 2020 statement [14]. Its protocol was registered prospectively in PROSPERO and can be obtained from the corresponding author. The completed PRISMA-DTA checklist appears in the Supplementary File (Supplementary Table S1), and the completed PRISMA 2020 checklist appears in Supplementary File S4.

### 2.2. Eligibility Criteria

Eligibility was structured around a diagnostic accuracy framework specifying population, index test, reference standard, and outcomes. We retained original primary diagnostic accuracy studies (cohort or diagnostic case–control) that (i) enrolled people of any age, sex, or clinical setting being assessed for any form of heart disease; (ii) tested an AI-based algorithm (machine learning, deep learning, or neural networks) applied to the 12-lead ECG; (iii) employed a valid reference standard—clinician-read 12-lead ECG, cardiac imaging by echocardiography or magnetic resonance, Holter or telemetry, invasive angiography, or accepted clinical criteria—in all or nearly all participants; and (iv) supplied enough information to build, or to reconstruct, a 2 × 2 contingency table (true positives [TP], false positives [FP], true negatives [TN], false negatives [FN]).

We excluded conference abstracts, editorials, reviews, case reports, and any work lacking a full text; publications not written in English; reports that described only signal- or segment-level performance without patient-level outcomes; and analyses that relied on ECG-derived features without an explicit AI algorithm. Reports lacking external or internal validation were appraised critically and earmarked for sensitivity analysis.

### 2.3. Information Sources and Search Strategy

PubMed, Scopus, Web of Science, and Embase were searched from their inception up to 31 March 2026. Each search blended controlled vocabulary (for example, MeSH) with free-text terms covering artificial intelligence, deep learning, electrocardiography, and cardiovascular disease, joined by Boolean operators; the complete strings appear in Supplementary File S1. To capture further eligible records, we manually screened the reference lists of the included studies and of pertinent reviews.

### 2.4. Study Selection

**Figure 1:**
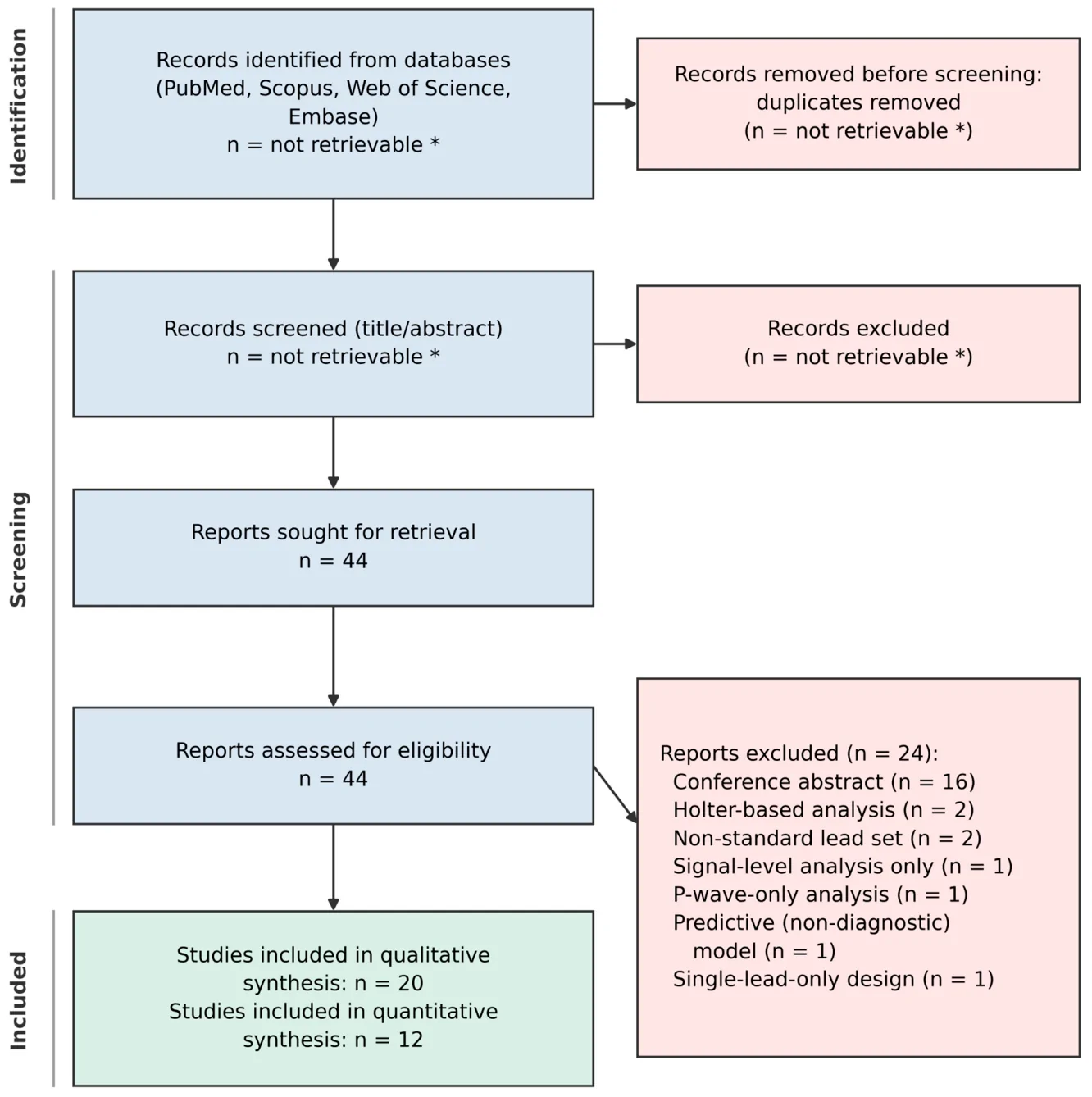
PRISMA-DTA flow diagram for study selection. * Record-level counts at the identification and title/abstract screening stages were not preserved in the screening log and could not be reconstructed retrospectively; this is reported as a protocol deviation (Supplementary File S2). The flow is complete from the stage of reports sought for retrieval onwards.

All records were uploaded to Rayyan, where duplicates were removed. Working independently, two reviewers (O.A.R.-T. and E.T.-A.) examined titles and abstracts and subsequently the full texts, settling disagreements through discussion or, where needed, arbitration by a third reviewer (J.J.B.). Figure 1 (the PRISMA-DTA flow diagram) outlines this selection process. Record-level counts at the identification and title/abstract screening stages were not preserved in the screening log and could not be reconstructed retrospectively; this is reported as a protocol deviation (Supplementary File S2), and the flow is complete and auditable from the stage of reports sought for retrieval onwards.

### 2.5. Data Extraction

Using a standardised, pilot-tested form, we recorded study descriptors (author, year, country, design), participant features (sample size, demographics, setting), characteristics of the index test (lead count, AI architecture, training and validation approach, decision threshold), the reference standard, the target condition and its threshold, and diagnostic performance (TP, FP, TN, FN, sensitivity, specificity, area under the curve, prevalence). Extraction was carried out independently by two reviewers, with discrepancies settled by consensus or third-party adjudication.

Where integer 2 × 2 cells were absent but sample size together with sensitivity/specificity (or PPV/NPV and prevalence) was available, we reconstructed the cells and logged them in the data dictionary (Supplementary Table S2). If a study presented several thresholds, models, or sub-cohorts, we pre-specified one primary row per study to prevent unit-of-analysis errors, treating the remaining sub-cohorts as secondary or exploratory analyses (Supplementary Table S3). Protocol deviations are detailed in Supplementary File S2.

### 2.6. Risk of Bias and Applicability

Risk of bias and applicability were judged independently by two reviewers with the QUADAS-2 tool [15], which spans four domains (patient selection, index test, reference standard, and flow and timing), and disagreements were settled by discussion. The resulting judgements are reported in Section 3.8, as per-study traffic-light plots and as a summary plot; the full signalling-question responses appear in Supplementary Table S4.

### 2.7. Statistical Analysis

For each study, sensitivity and specificity were calculated from the 2 × 2 cells with exact binomial 95% confidence intervals. The hierarchy of analyses was fixed in advance. The pre-specified primary synthesis was the condition-specific estimate for HF/LVSD. Other target conditions were synthesised separately, condition by condition. A cross-condition estimate that combines all eligible studies was computed as a secondary, descriptive summary only; because it aggregates targets whose electrophysiological substrate, prevalence, and reference standards differ, it is not intended as a clinically actionable operating characteristic and is reported as such throughout.

Whenever four or more eligible studies shared a target condition, a bivariate random-effects (Reitsma) model [16] modelled sensitivity and specificity jointly on the logit scale while accounting for their within-study correlation, and the matching HSROC curve was generated using the Rutter and Gatsonis parameterisation [17]. Pooled values, each with a 95% confidence interval, were produced for sensitivity, specificity, and the HSROC area under the curve (AUC). For any subgroup containing fewer than four studies, the bivariate model is not identifiable; in those cases, we report descriptive statistics (median and range) and, where an interval estimate was informative, a univariate DerSimonian–Laird random-effects pooling of logit-sensitivity and logit-specificity separately, labelled as such.

Handling of threshold variation: The included models did not share a common decision cut-off: some reported a probability threshold selected to maximise the Youden index in a development cohort, others a vendor-fixed operating point, and others a threshold chosen to achieve a target sensitivity. The bivariate model does not require a common threshold. Each study contributes a single (sensitivity, specificity) pair measured at whatever operating point that study used, and variation in those thresholds is absorbed by the random-effects structure: differences in implicit threshold generate a negative correlation between logit-sensitivity and logit-specificity across studies, which the bivariate model estimates explicitly as the between-study correlation ρ. The Rutter–Gatsonis HSROC reparameterisation makes the same information visible geometrically, since the fitted curve traces the accuracy attainable as the implicit threshold is varied, and the shape parameter β quantifies asymmetry in that trade-off. We therefore report ρ and β alongside every pooled estimate, interpret the summary point as the average operating point of the included models rather than as the performance of a single recommended cut-off, and read the HSROC curve—not the summary point—as the estimate that is comparable across studies with differing thresholds. To avoid mixing thresholds within a study, exactly one operating point per study was carried into the synthesis, selected in advance as described in Section 2.5 (Supplementary Table S3).

We probed heterogeneity by inspecting paired forest plots, by the scatter of study points around the HSROC curve, and through subgroup analyses defined by target condition, ECG modality (single- versus multi-lead), AI class (deep learning versus classical machine learning), and clinical setting. When at least 10 studies were available, small-study effects were assessed with the Deeks funnel-plot asymmetry test, based on the diagnostic odds ratio and effective sample size [18]. We note in advance that in DTA meta-analyses a significant Deeks test is not equivalent to evidence of publication bias: because the diagnostic odds ratio depends on the operating point, genuine between-study heterogeneity in accuracy or in threshold, and the inclusion of small studies with extreme diagnostic odds ratios, will produce asymmetry in the absence of any selective publication [18]. The test was therefore interpreted as a signal of small-study effects requiring explanation rather than as a bias estimate.

Pre-specified sensitivity analyses examined the influence of study quality and of study size. For risk of bias we (i) excluded all studies rated at high overall risk on QUADAS-2 and (ii) restricted the synthesis to studies rated at low overall risk. Because only three studies met the strict low-risk criterion, the restricted analysis was carried out with the univariate random-effects approach described above and is reported as exploratory. A further sensitivity analysis restricted the HF/LVSD synthesis to the four large external validation cohorts. The certainty of evidence was graded with the GRADE framework adapted for diagnostic tests [19]. We ran every analysis in R version 4.3 with the mada [20], meta, and metafor packages; Supplementary File S3 summarises the analytic pipeline, and the analytic code can be obtained from the corresponding author on reasonable request.

## 3. Results

### 3.1. Study Selection

The database searches yielded 44 full-text reports assessed for eligibility, of which 24 were excluded with documented reasons (16 conference abstracts, 2 Holter-based analyses, 2 using non-standard lead sets, 1 signal-only analysis, 1 P-wave-only analysis, 1 predictive [non-diagnostic] model, and 1 single-lead-only design unsuitable for our 2 × 2 reconstruction); 20 studies thus remained for qualitative synthesis, and 12 of these fed the quantitative synthesis. Supplementary Table S5 lists the exclusion reasons in full, and Figure 1 summarises the selection process.

### 3.2. Characteristics of Included Studies

Published between 2021 and 2026, the 20 included studies were carried out in nine countries across three continents (Table 1). Most adopted a retrospective diagnostic cohort or external validation cohort design (n = 13), accompanied by three prospective studies, three diagnostic case–control studies, and one case-only study. Sample sizes spanned 36 to 42,291 evaluable patients (155,442 in total). The conditions targeted comprised HF/LVSD or reduced left ventricular ejection fraction (k = 6 study-level entries; Adedinsewo 2024 [21], Carter 2026 [22], König 2024 [23], Lee 2025 [24], Thambiraj 2026 [25], Sun 2021 [26]), peripartum cardiomyopathy (Karabayir 2024 [27]), atrial fibrillation or atrial arrhythmia (Fiorina 2024 [28], Lueken 2025 [29]), coronary artery disease, acute coronary syndromes and ST-elevation myocardial infarction (Awasthi 2023 [30], Chang 2021 [31], Tang 2023 [32], Park 2023 [33], Díaz-Herrera 2025 [34], Luo 2026 [35]), hypertrophic cardiomyopathy (Siontis 2023 [36], Babur Guler 2026 [37]), left ventricular hypertrophy (Huang 2025 [38], Liu 2023 [39]), and acute pulmonary embolism (Valente Silva 2023 [40]). The index tests were chiefly deep neural networks—mostly convolutional, with attention-based or recurrent variants—applied to 12-lead, 8-lead, or single-lead tracings; a single study relied on classical machine learning (CatBoost) trained on engineered ECG features [38]. Reference standards ranged across echocardiography (Simpson method or LV mass index), cardiac magnetic resonance, coronary angiography, computed tomography pulmonary angiography, expert ECG adjudication, and ICD-coded diagnoses. Full study characteristics are given in Table 1, and per-study DTA data, including the 2 × 2 cells, in Table 2.

**Table 1:** Characteristics of the included studies (N = 20).

Study	Country	Design	N	Target Condition	ECG Modality	AI Model/Class	Reference Standard	Validation
Adedinsewo 2024 [21]	USA	Prospective cohort	100	LVEF < 50% (peripartum)	12-lead	Mayo AI-ECG CNN (DL)	Echocardiography	External/prospective
Awasthi 2023 [30]	USA	Case–control	10,110	Obstructive CAD	12-lead	CNN (DL)	Angiography/CT	Internal
Babur Guler 2026 [37]	Turkey	Case-only	681	HCM	12-lead (image)	ECG-Vision tools (DL)	Clinical/echo/CMR	External
Carter 2026 [22]	USA	Retrospective external validation	13,960	LVEF ≤ 40%	12-lead	Anumana ECG-AI LEF CNN (DL)	Echocardiography (Simpson)	External
Chang 2021 [31]	Taiwan	Retrospective	6037	STEMI + 12 rhythm classes	12-lead	Bi-LSTM (DL)	Cardiologist ECG label	Internal
Díaz-Herrera 2025 [34]	Mexico	Prospective derivation	36	ACOMI/OMI	12-lead (image)	InceptionResNetV2 (DL)	Coronary angiography	Internal
Fiorina 2024 [28]	France	Prospective cohort	393	Atrial arrhythmia	Single-lead (smartwatch)	Cardiologs DNN (DL)	Expert 12-lead ECG	External
Huang 2025 [38]	Taiwan	Retrospective cohort	8403	Left ventricular hypertrophy	12-lead (features)	CatBoost (ML)	Echo (LV mass index)	Internal
Karabayir 2024 [27]	USA	Case–control	115 + 43	Peripartum cardiomyopathy	12-lead	1D-CNN (DL)	ICD-coded diagnosis	Internal + external
König 2024 [23]	Germany	Retrospective external validation	42,291	LVEF < 40%	12-lead	Yagi CNN (DL)	Echocardiography (Simpson/triplane)	External
Lee 2025 [24]	USA	Retrospective external validation	22,599	LVEF < 40%	12-lead (image)	ECG Buddy (ARPI) (DL)	Echo (discharge-note LVEF)	External
Liu 2023 [39]	China	Two-site multi-label	9596	LVH (among 27 classes)	12-lead (image)	AA-ECG ResNet-34 (DL)	Expert ECG interpretation	External
Lueken 2025 [29]	Germany	Retrospective screening	224 (test)	Atrial fibrillation	Single-lead (MyDiagnostick)	Down-scaled CNN (DL)	Expert single-lead adjudication	Internal
Luo 2026 [35]	China	Retrospective cohort	125 (STEMI cohort)	STEMI/NSTEMI/UA/aortic dissection	12-lead (image)	CNN + attention (DL)	Final clinical diagnosis	Internal
Park 2023 [33]	South Korea	Retrospective cohort	723	Obstructive CAD (stable angina)	12-lead (image)	QCG (ARPI) ResNet (DL)	Invasive coronary angiography	Internal
Siontis 2023 [36]	USA	Case–control derivation	13,394	HCM	Single-lead (median-beat)	Median-beat CNN (DL)	Clinical criteria (echo/CMR)	Internal
Sun 2021 [26]	China	Retrospective cohort	2530	LVEF ≤ 50%	12-lead (image)	LeNet-5 CNN (DL)	Echocardiography (Simpson)	Internal
Tang 2023 [32]	China	Retrospective cohort	1054	CAD ≥ 50% stenosis	8-lead	SE-ResNet-50 (DL)	Coronary CT angiography	Internal
Thambiraj 2026 [25]	USA	Retrospective multicentre	22,925	LVEF ≤ 40%	12-lead	2D-CNN ECG-only (DL)	Echo (NLP-extracted)	Internal + external (cMRI)
Valente Silva 2023 [40]	Portugal	Retrospective cohort	103	Acute pulmonary embolism	12-lead	ResNet-18 + attention (DL)	CT pulmonary angiography	Internal

Studies are listed in alphabetical order by first author. Sample sizes refer to evaluable patients (or ECG–reference pairs) in the test set used for diagnostic accuracy. Fiorina 2024 [28] enrolled 393 participants, of whom 390 contributed evaluable ECG–reference pairs to the 2 × 2 table (Table 2). AI = artificial intelligence; CAD = coronary artery disease; CMR = cardiac magnetic resonance; CNN = convolutional neural network; DL = deep learning; DNN = deep neural network; HCM = hypertrophic cardiomyopathy; LVEF = left ventricular ejection fraction; LVH = left ventricular hypertrophy; ML = machine learning; STEMI = ST-elevation myocardial infarction; ACOMI = acute coronary occlusive myocardial infarction; OMI = occlusion myocardial infarction.

**Table 2:** Per-study diagnostic accuracy data for the quantitative synthesis (k = 12).

Study	Target Condition	TP	FP	FN	TN	Sensitivity (95% CI)	Specificity (95% CI)	AUC	QUADAS-2
Adedinsewo 2024 [21]	LVEF < 50%	6	0	0	94	1.00 (0.54–1.00)	1.00 (0.96–1.00)	1.00	Low
Carter 2026 [22]	LVEF ≤ 40%	926	2115	170	10,749	0.84 (0.82–0.87)	0.84 (0.83–0.84)	0.92	Low
König 2024 [23]	LVEF < 40%	5419	8148	1164	27,560	0.82 (0.81–0.83)	0.77 (0.77–0.78)	0.88	Low
Lee 2025 [24]	LVEF < 40%	2580	3759	441	15,819	0.85 (0.84–0.87)	0.81 (0.80–0.81)	0.91	High
Thambiraj 2026 [25]	LVEF ≤ 40%	2314	7240	189	13,182	0.92 (0.91–0.93)	0.65 (0.64–0.65)	0.88	High
Fiorina 2024 [28]	Atrial arrhythmia	122	13	12	243	0.91 (0.85–0.95)	0.95 (0.91–0.97)	—	Unclear
Lueken 2025 [29]	Atrial fibrillation	56	12	0	156	1.00 (0.94–1.00)	0.93 (0.88–0.96)	0.99	High
Tang 2023 [32]	CAD ≥ 50% stenosis	290	184	132	448	0.69 (0.64–0.73)	0.71 (0.67–0.74)	0.75	High
Luo 2026 (STEMI) [35]	STEMI	42	1	4	78	0.91 (0.79–0.98)	0.99 (0.93–1.00)	0.99	High
Siontis 2023 [36]	HCM	487	2046	122	10,739	0.80 (0.77–0.83)	0.84 (0.83–0.85)	0.90	High
Huang 2025 [38]	LVH	2603	819	615	4366	0.81 (0.79–0.82)	0.84 (0.83–0.85)	0.80	Unclear
Valente Silva 2023 [40]	Acute PE	19	0	19	65	0.50 (0.33–0.67)	1.00 (0.94–1.00)	0.75	Unclear

Studies that contributed reconstructable 2 × 2 tables and entered the analyses. Confidence intervals are exact binomial 95% CIs. The QUADAS-2 column gives the overall risk-of-bias judgement used in the sensitivity analyses reported in Section 3.9. AUC = area under the receiver operating characteristic curve; CAD = coronary artery disease; HCM = hypertrophic cardiomyopathy; LVEF = left ventricular ejection fraction; LVH = left ventricular hypertrophy; PE = pulmonary embolism; STEMI = ST-elevation myocardial infarction. 2 × 2 cells reconstructed when not directly reported (see Supplementary Table S2).

### 3.3. Pre-Specified Primary Synthesis: Heart Failure/Left Ventricular Systolic Dysfunction

**Figure 2:**
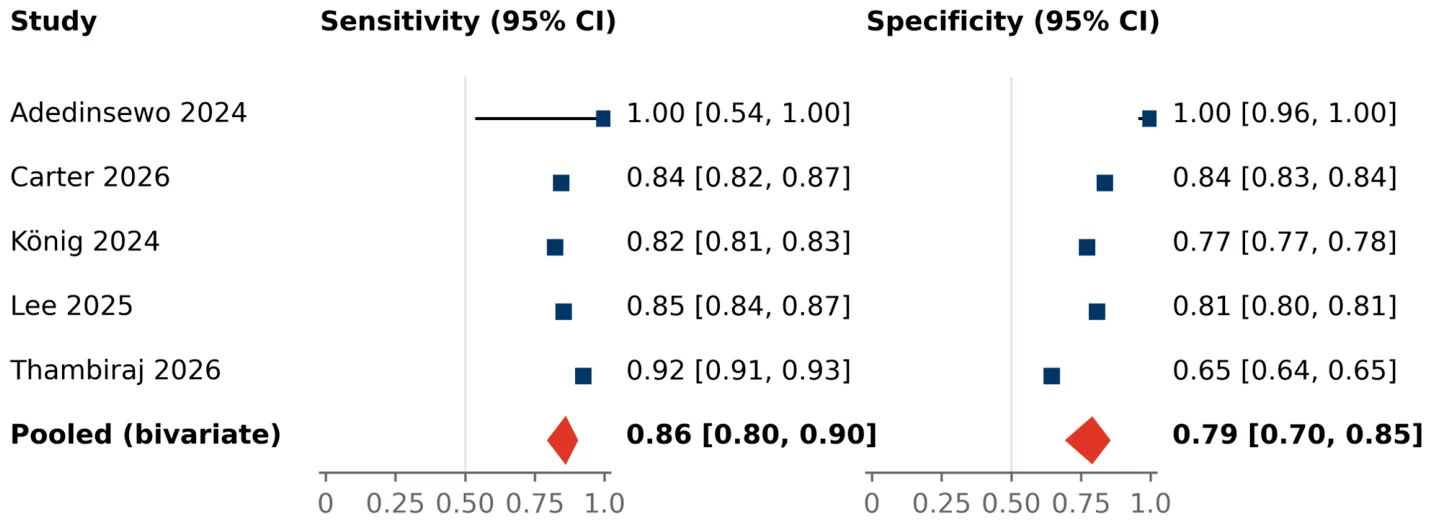
Paired forest plot of per-study sensitivity and specificity with exact binomial 95% confidence intervals for the pre-specified primary synthesis: heart failure/left ventricular systolic dysfunction (HF/LVSD; k = 5). Diamonds show the bivariate random-effects (Reitsma) summary estimates. Studies: Adedinsewo 2024 [21], Carter 2026 [22], König 2024 [23], Lee 2025 [24] and Thambiraj 2026 [25].

**Figure 3:**
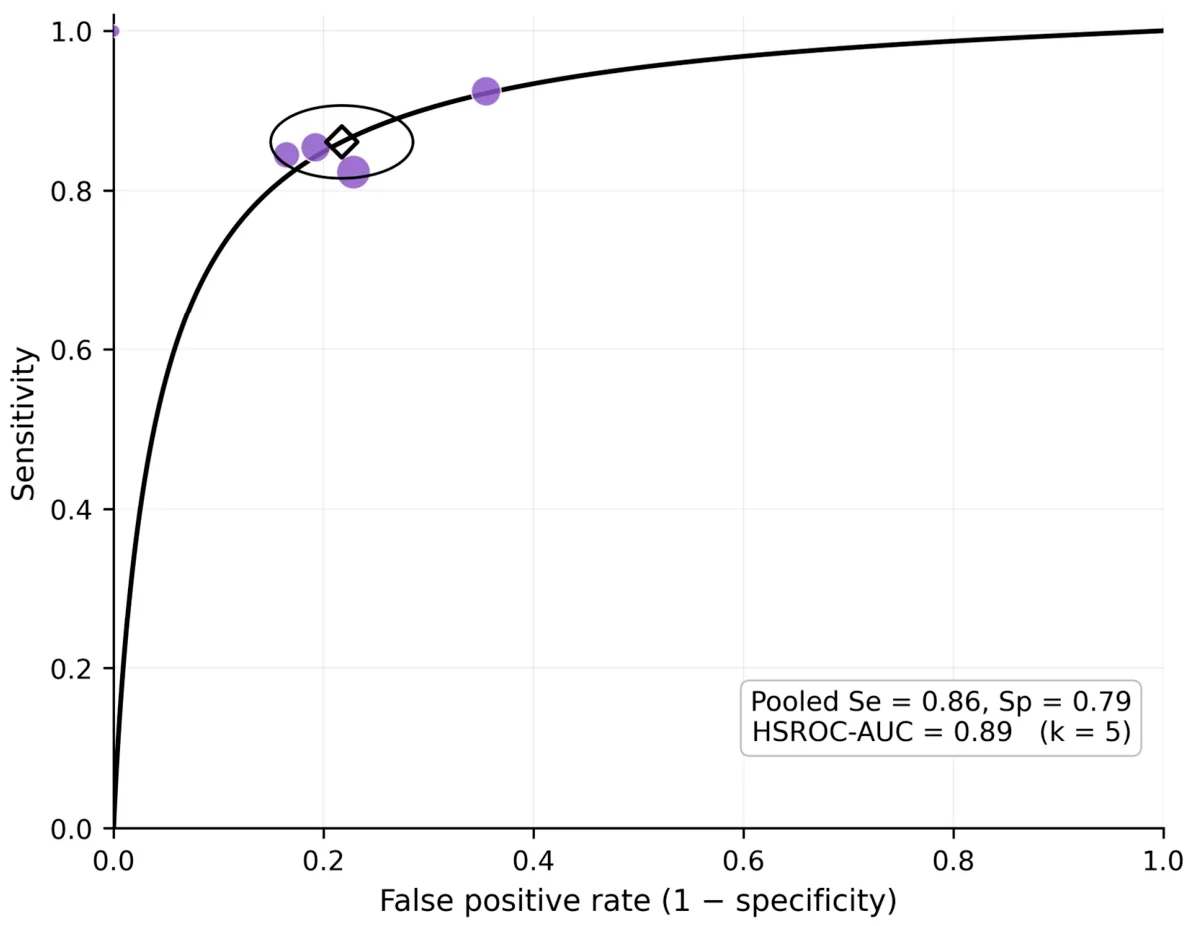
Hierarchical summary receiver operating characteristic (HSROC) curve for the HF/LVSD subgroup (k = 5). Circle area is proportional to study size; the open diamond marks the summary operating point and the ellipse its 95% confidence region. Because the included models used different decision thresholds, the curve rather than the summary point is the quantity comparable across studies. Studies: Adedinsewo 2024 [21], Carter 2026 [22], König 2024 [23], Lee 2025 [24] and Thambiraj 2026 [25].

Five external validation studies enrolling 101,875 patients with paired ECG–echocardiography data evaluated AI-ECG for the detection of low LVEF (≤40% or <50%): Adedinsewo 2024 [21], Carter 2026 [22], König 2024 [23], Lee 2025 [24], and Thambiraj 2026 [25]. The pooled sensitivity was 0.86 (95% CI, 0.80–0.90) and the pooled specificity 0.79 (95% CI, 0.70–0.85), with an HSROC AUC of 0.89 (Table 3; Figure 2 and Figure 3). Disease prevalence in the test sets ranged from 6.0% (Adedinsewo 2024) to 15.6% (König 2024). Performance was consistent across the large external validation cohorts (Carter 2026: sensitivity 0.84, specificity 0.84; König 2024: 0.82 and 0.77; Lee 2025: 0.85 and 0.81), while Thambiraj 2026 showed higher sensitivity (0.92) but lower specificity (0.65), reflecting a probability-threshold trade-off. Adedinsewo 2024 (n = 100, 6 cases) returned perfect classification but with wide confidence intervals, consistent with its small case count [21]. This subgroup is the only one in which pooling rests on several independent external validations of comparable models against the same imaging reference standard, and it is accordingly the estimate we regard as clinically interpretable.

### 3.4. Other Target Conditions: Condition-Specific Synthesis

**Figure 4:**
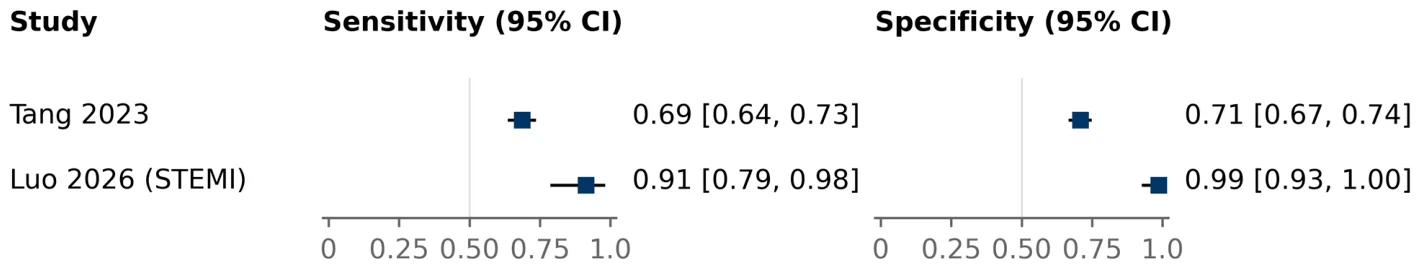
Paired forest plot for the coronary artery disease/acute coronary syndromes (CAD/ACS-MI) subgroup (k = 2). With fewer than four studies the bivariate model was not fitted and no pooled estimate is shown; per-study estimates are presented with exact binomial 95% confidence intervals. Studies: Tang 2023 [32] and Luo 2026 (STEMI cohort) [35].

Two atrial fibrillation studies entered the descriptive synthesis (Fiorina 2024 [28], Lueken 2025 [29]), giving a median sensitivity of 0.96 (range 0.91–1.00) and a median specificity of 0.94 (range 0.93–0.95). For coronary artery disease and acute coronary syndromes/myocardial infarction (CAD/ACS-MI), two studies qualified (Tang 2023 [32] and the Luo 2026 STEMI cohort [35]), and their estimates diverged widely (median sensitivity 0.80, range 0.69–0.91; median specificity 0.85, range 0.71–0.99) (Figure 4). Hypertrophic cardiomyopathy (Siontis 2023 [36]) showed a sensitivity and specificity of 0.80 and 0.84, and left ventricular hypertrophy (Huang 2025 [38]) of 0.81 and 0.84. The one eligible acute pulmonary embolism study (Valente Silva 2023 [40]) combined high specificity (1.00) with low sensitivity (0.50). In none of these conditions did the number of eligible studies reach the pre-specified threshold of four, so no pooled estimate is offered for them; the condition-level summaries in Table 3 are descriptive. Sun 2021 [26], Park 2023 [33], Chang 2021 [31], and Babur Guler 2026 [37] offered no recoverable 2 × 2 data (absent denominators, absent specificity, an expert-ECG circular reference, or a case-only design) and so were kept only for narrative purposes, as was Liu 2023 [39], whose left ventricular hypertrophy label was assigned by expert ECG interpretation within a 27-class multi-label task and yielded no target-specific 2 × 2 table. Karabayir 2024 [27], Awasthi 2023 [30], and Díaz-Herrera 2025 [34] were dropped owing to case–control designs with artefactual prevalence. These eight exclusions account for the difference between the 20 studies reviewed and the 12 pooled (Supplementary Table S3).

### 3.5. Secondary, Descriptive Cross-Condition Summary

**Figure 5:**
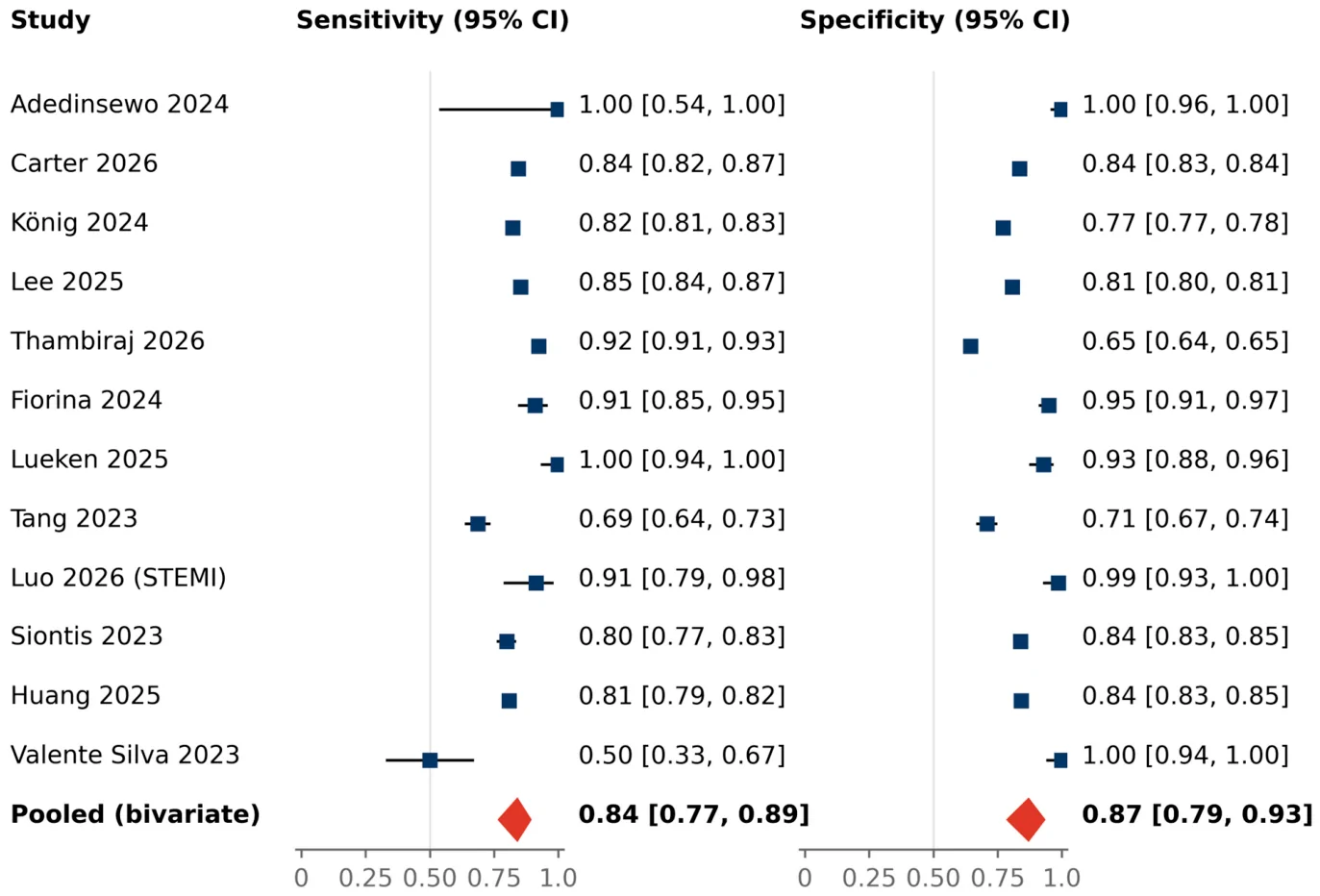
Paired forest plot of per-study sensitivity and specificity for the secondary, descriptive cross-condition synthesis (k = 12). The pooled diamonds aggregate heterogeneous target conditions and are provided for descriptive purposes only; the condition-specific estimates (Figure 2, Table 3) are the clinically interpretable results. Studies: the 12 studies listed in Table 2 [21,22,23,24,25,28,29,32,35,36,38,40].

**Figure 6:**
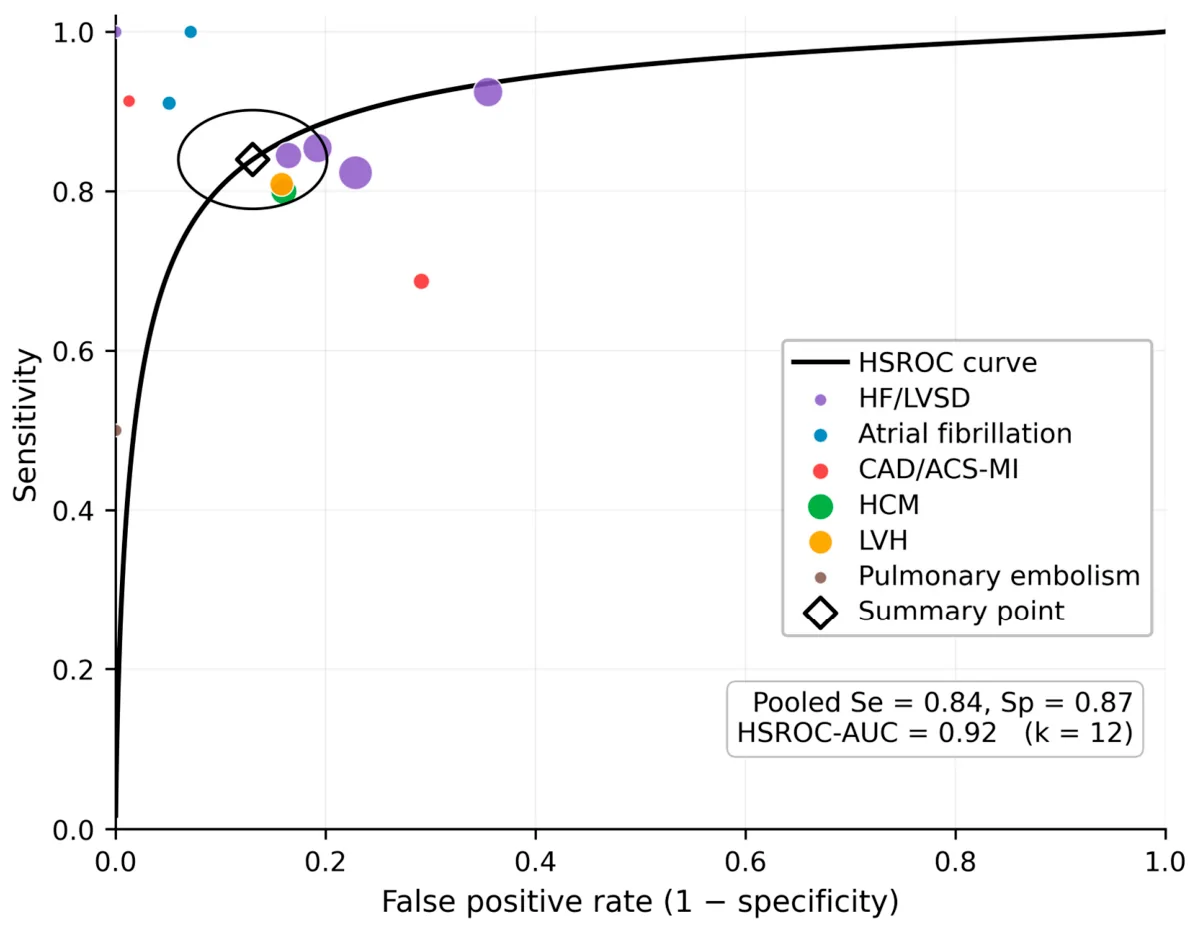
HSROC curve for the secondary cross-condition analysis (k = 12). Point colour denotes the target condition and circle area is proportional to study size. The wide scatter across conditions illustrates why this summary is reported descriptively rather than as a clinical operating characteristic. Studies: the 12 studies listed in Table 2 [21,22,23,24,25,28,29,32,35,36,38,40].

Twelve studies (125,568 ECG–reference pairs) provided reconstructable 2 × 2 tables (Table 2; Figure 5). Fitted across all target conditions, the bivariate model returned a sensitivity of 0.84 (95% CI, 0.77–0.89) and a specificity of 0.87 (95% CI, 0.79–0.93), with an HSROC AUC of 0.92 (Table 3; Figure 6). The paired forest plot and the HSROC space revealed pronounced between-study heterogeneity: most large external validation studies grouped near the upper-left quadrant, whereas smaller single-centre studies gave more scattered estimates. We emphasise that this figure aggregates conditions whose electrophysiological substrate, prevalence, and reference standards are not exchangeable—atrial fibrillation, in which the arrhythmia is directly represented in the tracing, sits alongside pulmonary embolism, in which the ECG signal is indirect—and that the resulting summary point corresponds to no clinical decision that any individual patient faces. It is reported for completeness and comparability with earlier cross-condition reviews, and it should not be quoted as the accuracy of AI-ECG for any single indication. The condition-specific estimates in Section 3.3 and Section 3.4 are the clinically interpretable results of this review.

### 3.6. Threshold Effects and Heterogeneity

Threshold variation was a substantial and quantifiable source of heterogeneity. In the HF/LVSD synthesis the estimated between-study correlation between logit-sensitivity and logit-specificity was −0.86 (equivalently +0.86 between logit-sensitivity and the logit false-positive rate), with between-study standard deviations of 0.38 and 0.40 on the logit scale and an HSROC shape parameter β of 0.06, indicating a nearly symmetric trade-off. A correlation of this magnitude is the statistical signature of a strong implicit threshold effect: the five HF/LVSD models are, to a good approximation, sitting at different points on a common accuracy curve rather than differing in intrinsic discrimination. This is consistent with what the studies report—Thambiraj 2026 [25] operated at a deliberately sensitivity-favouring cut-off (0.92/0.65) while König 2024 [23] operated nearer the Youden point (0.82/0.77)—and it is the reason the HSROC curve, rather than the summary point, is the appropriate basis for cross-study comparison in this subgroup. In the secondary cross-condition analysis the correlation was near zero (+0.08) with much larger between-study standard deviations (0.67 and 0.86), indicating that variability there is driven predominantly by differences in the target condition itself rather than by threshold—a further reason not to read that estimate as a single operating characteristic.

### 3.7. Small-Study Effects

**Figure 7:**
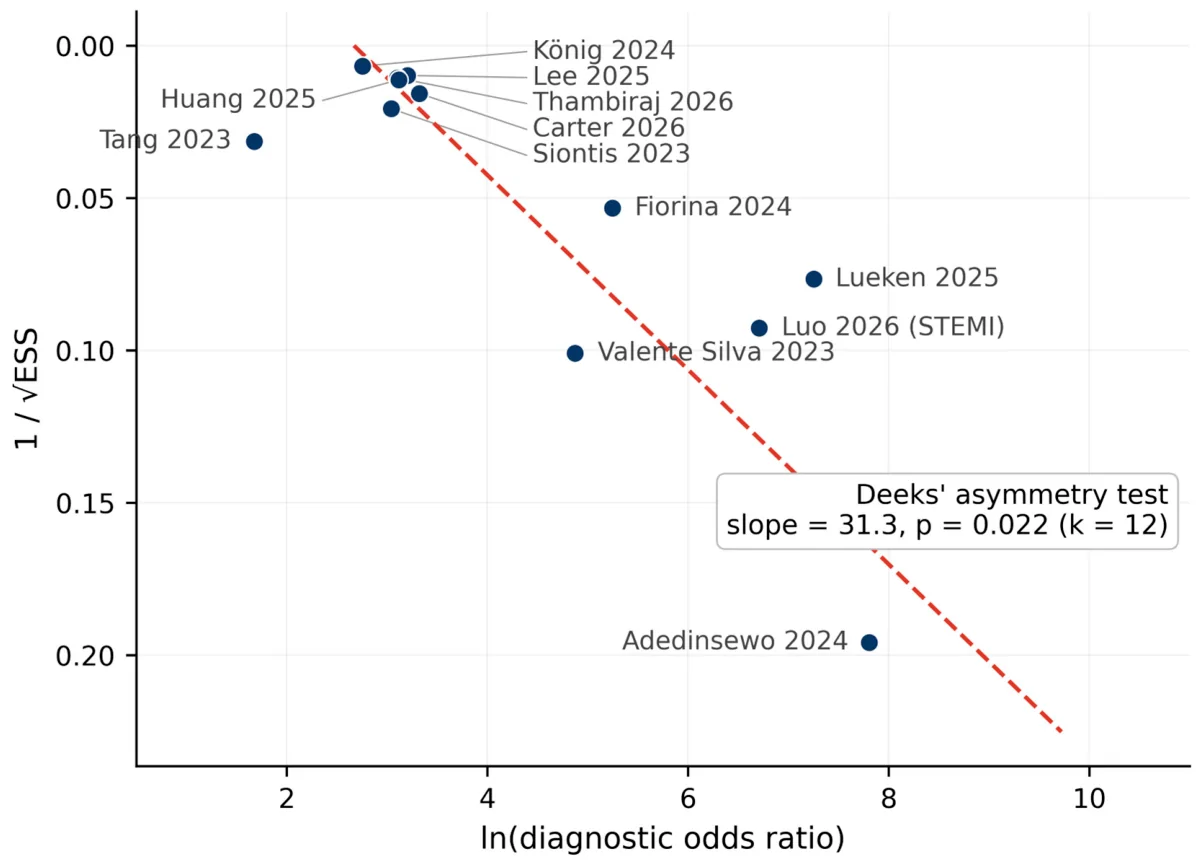
Deeks funnel plot and asymmetry test for the cross-condition synthesis (k = 12). ESS = effective sample size. Asymmetry in a diagnostic test accuracy funnel plot is produced by heterogeneity in true accuracy and in decision threshold as readily as by selective publication and is therefore reported here as a small-study effect, not as evidence of publication bias. Studies: the 12 studies listed in Table 2 [21,22,23,24,25,28,29,32,35,36,38,40].

With 12 studies eligible for the cross-condition synthesis, the Deeks funnel-plot asymmetry test was significant (slope = 31.3; *p* = 0.022) (Figure 7). As set out in Section 2.7, this should not be read as demonstrating publication bias. The Deeks test regresses the log diagnostic odds ratio on the inverse root of the effective sample size, so it responds to any process that makes small studies differ systematically from large ones—including genuine between-study heterogeneity in accuracy and in operating point, both of which are present here. The asymmetry in our sample is driven principally by two small studies at the extremes of the diagnostic odds ratio distribution: Adedinsewo 2024 [21], with n = 100, six cases and perfect classification, and Valente Silva 2023 [40], with n = 103 and a specificity of 1.00 at a sensitivity of 0.50. Removing Adedinsewo 2024 from the HF/LVSD synthesis changed the pooled estimates only marginally (Section 3.9), which is what would be expected if the asymmetry reflects the behaviour of small studies with extreme estimates rather than the selective non-publication of negative results. We therefore report funnel asymmetry as an unexplained small-study effect that lowers confidence in the secondary cross-condition estimate, and we do not claim to have detected publication bias.

### 3.8. Risk of Bias

**Figure 8:**
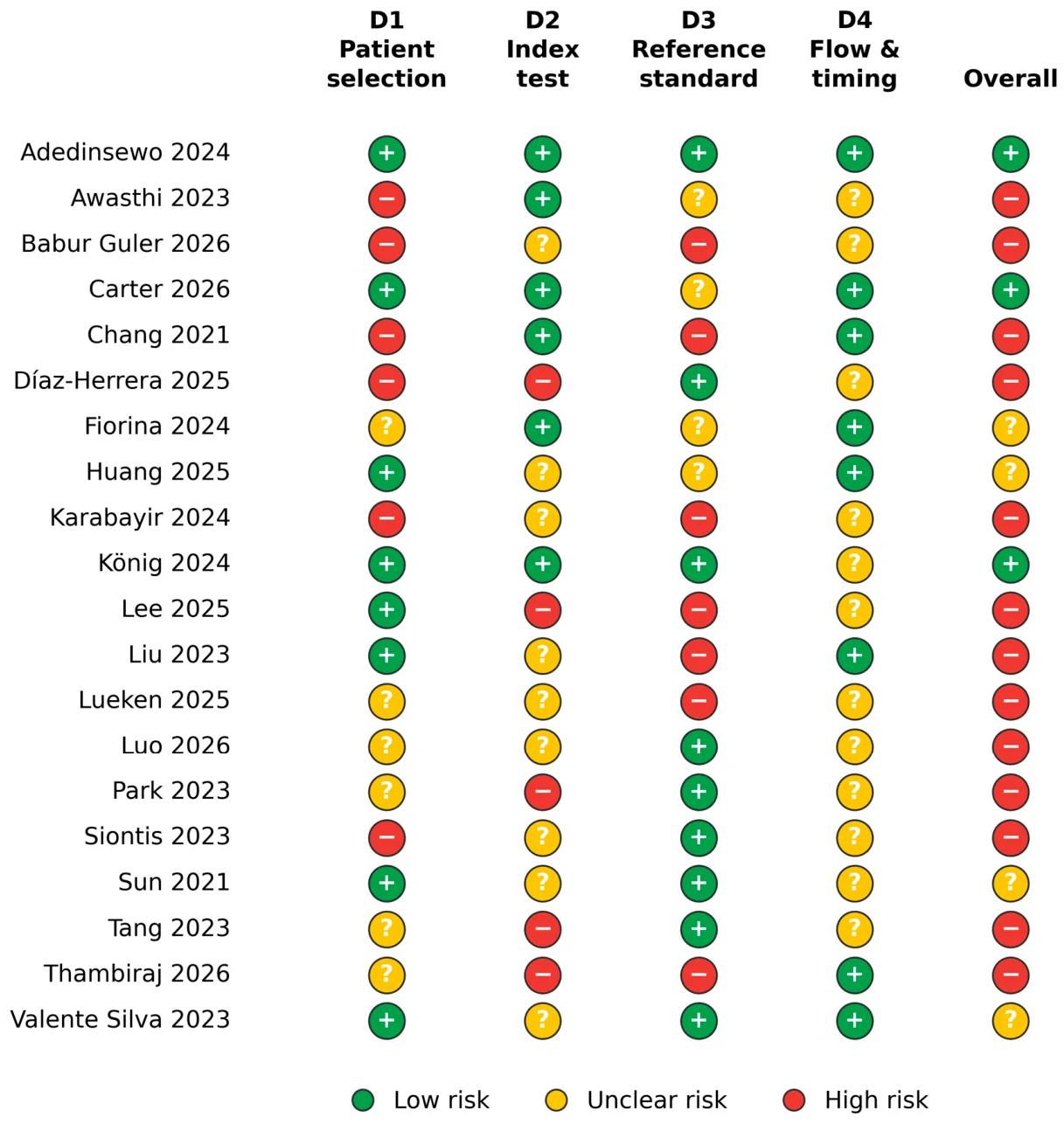
QUADAS-2 traffic-light plot of risk-of-bias judgements per study and domain (N = 20). D1, patient selection; D2, index test; D3, reference standard; D4, flow and timing. Studies: the 20 studies listed in Table 1 [21,22,23,24,25,26,27,28,29,30,31,32,33,34,35,36,37,38,39,40].

**Figure 9:**
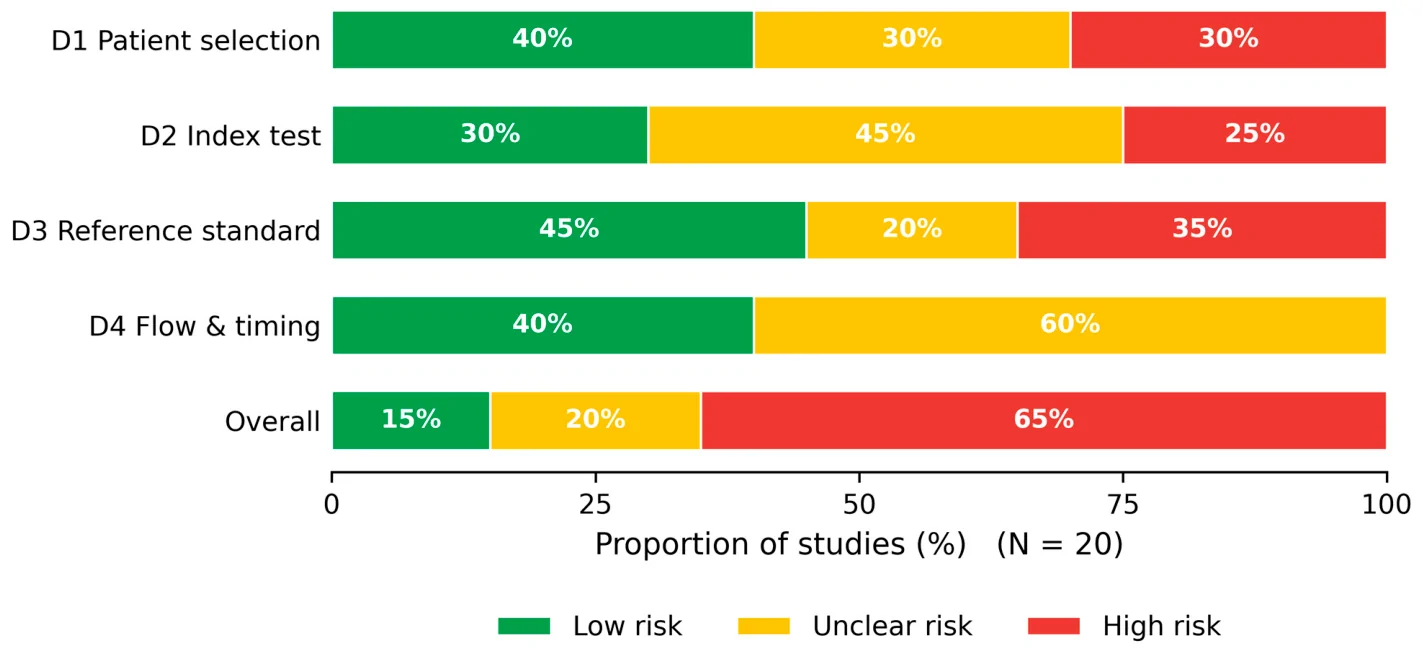
QUADAS-2 summary plot of risk-of-bias judgements across the 20 included studies, expressed as the proportion of studies in each judgement category per domain. Studies: the 20 studies listed in Table 1 [21,22,23,24,25,26,27,28,29,30,31,32,33,34,35,36,37,38,39,40].

Of the 20 included studies, 3 (15%) carried an overall low risk of bias, 4 (20%) were unclear, and 13 (65%) were judged high risk (Figure 8 and Figure 9; Table 4). Concerns were distributed across all four domains: the index test domain was rated high or unclear in 14/20 studies, patient selection and flow and timing in 12/20 each, and the reference standard in 11/20. The reference standard nonetheless attracted more judgements of outright high risk (7/20) than any other domain, stemming from reliance on expert ECG interpretation as truth (e.g., Chang 2021 [31], Liu 2023 [39], Fiorina 2024 [28]), retrospective harvesting of imaging results from electronic health records without independent re-adjudication (Carter 2026 [22], Lee 2025 [24], Thambiraj 2026 [25]), or ICD-coded outcomes (Karabayir 2024 [27]). Concerns over patient selection arose largely from case–control sampling that produced artefactual prevalence (e.g., Awasthi 2023 [30]) or case-only cohorts (Babur Guler 2026 [37]); no study was rated at high risk in the flow-and-timing domain, where the concern was almost always unclear reporting of the interval between the ECG and the reference test. Applicability concerns followed the same pattern and are summarised in Supplementary Table S4.

**Table 4:** QUADAS-2 risk-of-bias judgements per study and domain (N = 20).

Study	D1 Patient Selection	D2 Index Test	D3 Reference Standard	D4 Flow & Timing	Overall
Adedinsewo 2024 [21]	Low	Low	Low	Low	Low
Awasthi 2023 [30]	High	Low	Unclear	Unclear	High
Babur Guler 2026 [37]	High	Unclear	High	Unclear	High
Carter 2026 [22]	Low	Low	Unclear	Low	Low
Chang 2021 [31]	High	Low	High	Low	High
Díaz-Herrera 2025 [34]	High	High	Low	Unclear	High
Fiorina 2024 [28]	Unclear	Low	Unclear	Low	Unclear
Huang 2025 [38]	Low	Unclear	Unclear	Low	Unclear
Karabayir 2024 [27]	High	Unclear	High	Unclear	High
König 2024 [23]	Low	Low	Low	Unclear	Low
Lee 2025 [24]	Low	High	High	Unclear	High
Liu 2023 [39]	Low	Unclear	High	Low	High
Lueken 2025 [29]	Unclear	Unclear	High	Unclear	High
Luo 2026 [35]	Unclear	Unclear	Low	Unclear	High
Park 2023 [33]	Unclear	High	Low	Unclear	High
Siontis 2023 [36]	High	Unclear	Low	Unclear	High
Sun 2021 [26]	Low	Unclear	Low	Unclear	Unclear
Tang 2023 [32]	Unclear	High	Low	Unclear	High
Thambiraj 2026 [25]	Unclear	High	High	Low	High
Valente Silva 2023 [40]	Low	Unclear	Low	Low	Unclear

Domains are scored as Low, Unclear, or High risk of bias. Applicability judgements are summarised in Supplementary Table S4.

### 3.9. Sensitivity Analyses by Risk of Bias

Because two-thirds of the included studies were rated at high overall risk of bias, we repeated the syntheses after excluding them (Table 5). In the HF/LVSD subgroup, removing the two high-risk studies (Lee 2025 [24] and Thambiraj 2026 [25]) left three studies, below the threshold for the bivariate model; univariate random-effects pooling of these gave a sensitivity of 0.83 (95% CI, 0.81–0.85) and a specificity of 0.82 (95% CI, 0.75–0.87), compared with 0.86 and 0.79 in the primary analysis. Sensitivity was therefore marginally lower and specificity marginally higher, with confidence intervals that overlap the primary estimates throughout; the direction of the shift is what the threshold structure predicts, since the two excluded studies were the ones operating at the most sensitivity-favouring cut-offs. Notably, heterogeneity in sensitivity fell sharply once the high-risk studies were removed (I^2^ 97% to 42%), while heterogeneity in specificity did not, indicating that reference-standard quality and threshold choice act on the two dimensions differently.

Because the only three studies rated at low overall risk of bias across the whole review (Adedinsewo 2024 [21], Carter 2026 [22], König 2024 [23]) all address HF/LVSD, the strict low-risk-only analysis requested for the review as a whole is numerically identical to the HF/LVSD low-risk analysis just described. This coincidence is itself informative: within the current literature, the only body of AI-ECG evidence that satisfies QUADAS-2 across all four domains concerns the detection of reduced ejection fraction. Excluding high-risk studies from the secondary cross-condition analysis left six studies and gave a sensitivity of 0.80 (95% CI, 0.69–0.88) and a specificity of 0.92 (95% CI, 0.78–0.98) with an HSROC AUC of 0.91, again with wide, overlapping intervals. Finally, confining the HF/LVSD synthesis to the four large external validation studies (Carter 2026; König 2024; Lee 2025; Thambiraj 2026) produced a pooled sensitivity of 0.86 (95% CI, 0.78–0.92) and a pooled specificity of 0.77 (95% CI, 0.69–0.84), essentially identical to the primary estimate. Across every sensitivity analysis the HF/LVSD estimate stayed within approximately three percentage points of the primary result for both sensitivity and specificity.

### 3.10. Certainty of Evidence

Certainty for the HF/LVSD subgroup was graded moderate, downgraded for reference-standard-related risk of bias in some studies (Table 6); the robustness of this estimate to the exclusion of high-risk studies (Section 3.9; Supplementary Table S6) was taken into account and prevented a further downgrade for risk of bias. Certainty for the secondary cross-condition estimate was graded low on account of inconsistency, indirectness, and unexplained small-study effects (Table 6).

## 4. Discussion

This review set out to establish what can and cannot currently be claimed about the diagnostic accuracy of AI-ECG, and the answer is sharply asymmetric across conditions. For the detection of reduced ejection fraction the evidence base is now mature enough to support a pooled estimate—five independent external validations, more than 100,000 paired ECG–echocardiography observations, and an estimate that moves by no more than a few percentage points when the studies at highest risk of bias are removed. For every other cardiac target, the evidence remains a collection of single studies, and the honest summary is a range rather than a point.

Comparison with the wider literature: Our conclusions sit alongside, but are not interchangeable with, the recent reviews of this field. Lin and colleagues, surveying AI-ECG from research to clinical application, emphasise a translational trajectory in which the strongest evidence concerns asymptomatic low ejection fraction and paroxysmal atrial fibrillation during sinus rhythm, and in which randomised evaluations have begun to demonstrate workflow benefit rather than discrimination alone [10]. Our quantitative results are consistent with that reading and give it a numerical anchor: the two domains they highlight are precisely the two in which we found either a stable pooled estimate (HF/LVSD) or uniformly high per-study accuracy (atrial fibrillation). The reviews of AI in atherosclerotic cardiovascular disease [11] and in coronary intervention [12] converge on a different and equally important theme—that the principal obstacles are data bias, interpretability, and the absence of multicentre validation rather than model performance per se. Our QUADAS-2 results substantiate that claim empirically for the diagnostic setting: two-thirds of the studies we appraised were at high risk of bias, and the dominant reason was not the algorithm but the reference standard against which it was judged.

The comparison with Kany and colleagues is particularly instructive for the coronary domain, where our own pooled results were least stable [41]. Training an ECG-based model on more than 760,000 tracings and evaluating it in three external cohorts, they reported areas under the curve for prevalent coronary artery disease of 0.75–0.78—materially lower than the 0.88–0.92 typical of the ejection-fraction models in our HF/LVSD subgroup, and obtained with far larger samples than any coronary study we were able to include. That two lines of evidence, ours from small heterogeneous DTA studies and theirs from a large single-programme development and validation effort, arrive at the same conclusion strengthens the inference considerably: the electrocardiographic substrate for chronic coronary disease is genuinely weaker than that for ventricular dysfunction, and the divergent per-study estimates we observed for CAD/ACS-MI (0.69–0.91 sensitivity) most likely reflect differences in case mix and threshold rather than differences between models. Their finding that the model nonetheless stratified risk of subsequent myocardial infarction and heart failure also points to a distinction that DTA reviews such as ours cannot capture, namely that an ECG-derived score may carry prognostic information even where its cross-sectional diagnostic accuracy is modest.

Why the reference standard dominates: Nearly two-thirds of the included studies were rated high risk of bias on QUADAS-2. The index test domain drew concern in the largest number of studies (14/20 rated high or unclear), but the reference standard produced more judgements of outright high risk than any other domain (7/20), and this is not a peripheral observation. Treating expert ECG adjudication as truth (Chang 2021 [31], Liu 2023 [39], and partly Fiorina 2024 [28]) creates circularity when the index test is itself an ECG-derived classifier: what is measured is agreement with expert reading, not detection of anatomical or functional disease, and an algorithm can score highly on the former while adding nothing to the latter. Conversely, drawing imaging-based outcomes retrospectively from electronic health records or clinical narratives (Carter 2026 [22], Lee 2025 [24], Thambiraj 2026 [25]) is efficient and permits the very large samples that make external validation feasible, but it forgoes independent re-adjudication and inherits whatever misclassification the source records contain. Diagnostic case–control designs (Awasthi 2023 [30], Karabayir 2024 [27]) raise prevalence artificially and can distort sensitivity, specificity, and predictive values. These are solvable problems, and the three low-risk studies in our review demonstrate that they are solvable in practice.

Interpreting threshold variation and funnel asymmetry: Two features of our analysis deserve emphasis because they are easily over-read. First, the models we synthesised did not share a decision cut-off, and the strongly negative between-study correlation we estimated for HF/LVSD (ρ = −0.86) shows that this mattered. The bivariate and HSROC framework is designed for exactly this situation and does not require a common threshold, but it changes what the summary point means: it is the average operating point of the models as deployed, not the performance of a recommended cut-off, and the HSROC curve is the quantity that should be compared across studies. Reporting a single sensitivity/specificity pair without the accompanying curve would overstate the precision with which any future implementer could expect to reproduce it. Second, the significant Deeks test (*p* = 0.022) is best understood as a small-study effect rather than as publication bias. In DTA meta-analysis, asymmetry of the diagnostic-odds-ratio funnel is produced by heterogeneity in true accuracy and in threshold as readily as by selective publication, and the test has limited power to distinguish them [18]. In our sample the asymmetry is traceable to two small studies with extreme diagnostic odds ratios, and removing the most extreme of them barely moved the HF/LVSD estimate. We have therefore used the result to temper confidence in the cross-condition summary, not to assert that negative studies are missing.

Strengths and limitations: We defined the diagnostic accuracy framework and the statistical plan in advance, designated the HF/LVSD question as primary before analysis, reported according to PRISMA-DTA, applied QUADAS-2 in duplicate, and fitted a bivariate random-effects model with HSROC visualisation. We deliberately chose to prevent double-counting by pre-specifying a single primary row per study, to document every data reconstruction, and to confine ourselves to descriptive synthesis where pooling was inappropriate. The limitations are equally clear. The restriction to English-language reports may have excluded eligible work. Several 2 × 2 tables had to be reconstructed from reported summary measures. Record-level counts for the identification and screening stages of the search were not preserved and are reported as a protocol deviation. Most importantly, for every condition other than HF/LVSD the number of eligible studies was too small to pool, so our statements about those conditions rest on single estimates and should be treated as provisional. Because eligible papers may have appeared after the final search date, the protocol provides for an update.

Implications: In HF/LVSD, the current evidence positions AI-ECG as a complementary rule-out test in primary care and emergency settings, where a negative result might postpone further imaging in low-risk patients; even so, its positive predictive value stays modest at population-level prevalence, and confirmation still depends on downstream echocardiography [22]. For the remaining conditions, condition-specific prospective external validation against guideline-based reference standards would do more to advance the field than further model development. Reporting according to the TRIPOD and CONSORT-AI frameworks [42,43], and the inclusion of complete 2 × 2 confusion matrices at the pre-specified operating point in the main manuscript rather than the supplement, would substantially improve the feasibility of future meta-analytic synthesis; in our own review, five otherwise eligible studies could not be pooled because their 2 × 2 cells were unrecoverable—missing denominators, an unreported specificity, a circular expert-ECG reference standard, or a case-only design—and three more because case–control sampling made prevalence, and hence the cells, uninterpretable.

## 5. Conclusions

AI-based analysis of the ECG achieves moderately high diagnostic accuracy across a range of heart diseases, and the most consistent and reproducible evidence supports its use for detecting HF/LVSD against echocardiography; this estimate was robust to the exclusion of studies at high risk of bias. For the other target conditions examined, the available studies were too few to pool, and certainty is further tempered by methodological heterogeneity, a preponderance of retrospective designs, and concerns over the reference standard.

Several directions appear likely to strengthen the evidence base. Prospective external validation with independently adjudicated reference standards would address the domain in which we found risk of bias to be most concentrated. Transparent reporting of the complete 2 × 2 table at a pre-specified operating point, alongside the discrimination metrics that are usually reported, would make future studies meta-analysable—an issue that materially constrained the present review. Given the threshold heterogeneity we observed, reporting performance across a range of operating points, rather than at a single development-derived cut-off, would also help implementers understand what to expect in their own setting. We offer these as priorities suggested by the present evidence rather than as prescriptions, and we recognise that the appropriate balance between them will depend on the target condition and the deployment context.

## Figures and Tables

**Table 3:** Diagnostic accuracy by target condition.

Analysis	Role	Studies (k)	ECG–Reference Pairs	Sensitivity (95% CI or Range)	Specificity (95% CI or Range)	HSROC AUC
Heart failure/LVSD (bivariate)	Primary	5	101,875	0.86 (0.80–0.90)	0.79 (0.70–0.85)	0.89
Atrial fibrillation (descriptive)	Condition-specific	2	614	Median 0.96 (0.91–1.00)	Median 0.94 (0.93–0.95)	—
CAD/ACS-MI (descriptive)	Condition-specific	2	1179	Median 0.80 (0.69–0.91)	Median 0.85 (0.71–0.99)	—
Hypertrophic cardiomyopathy	Condition-specific	1	13,394	0.80	0.84	—
Left ventricular hypertrophy	Condition-specific	1	8403	0.81	0.84	—
Acute pulmonary embolism	Condition-specific	1	103	0.50	1.00	—
All conditions combined (bivariate)	Secondary, descriptive	12	125,568	0.84 (0.77–0.89)	0.87 (0.79–0.93)	0.92

Bivariate random-effects (Reitsma) estimates are reported when k ≥ 4; otherwise, medians and ranges from the per-study estimates are presented and no confidence interval is given. The cross-condition row combines heterogeneous target conditions and is provided as a descriptive summary only; it is not a clinically actionable operating characteristic (see Section 3.5). CAD/ACS-MI = coronary artery disease/acute coronary syndromes/myocardial infarction; CI = confidence interval; HSROC = hierarchical summary receiver operating characteristic; LVSD = left ventricular systolic dysfunction.

**Table 5:** Sensitivity analyses by risk of bias and study size.

Analysis	Studies Included	k	ECG–Reference Pairs	Model	Sensitivity (95% CI)	Specificity (95% CI)	HSROC AUC
HF/LVSD—primary analysis	Adedinsewo 2024 [21]; Carter 2026 [22]; König 2024 [23]; Lee 2025 [24]; Thambiraj 2026 [25]	5	101,875	Bivariate	0.86 (0.80–0.90)	0.79 (0.70–0.85)	0.89
HF/LVSD—excluding studies at high risk of bias	Adedinsewo 2024 [21]; Carter 2026 [22]; König 2024 [23]	3	56,351	Univariate ᵃ	0.83 (0.81–0.85)	0.82 (0.75–0.87)	NE
HF/LVSD—large external validation cohorts only	Carter 2026 [22]; König 2024 [23]; Lee 2025 [24]; Thambiraj 2026 [25]	4	101,775	Bivariate	0.86 (0.78–0.92)	0.77 (0.69–0.84)	0.89
All conditions—secondary analysis	All 12 studies in Table 2 [21,22,23,24,25,28,29,32,35,36,38,40]	12	125,568	Bivariate	0.84 (0.77–0.89)	0.87 (0.79–0.93)	0.92
All conditions—excluding studies at high risk of bias	Adedinsewo 2024 [21]; Carter 2026 [22]; König 2024 [23]; Fiorina 2024 [28]; Huang 2025 [38]; Valente Silva 2023 [40]	6	65,247	Bivariate	0.80 (0.69–0.88)	0.92 (0.78–0.98)	0.91
All conditions—restricted to studies at low risk of bias ᵇ	Adedinsewo 2024 [21]; Carter 2026 [22]; König 2024 [23]	3	56,351	Univariate ᵃ	0.83 (0.81–0.85)	0.82 (0.75–0.87)	NE

ᵃ With fewer than four studies the bivariate model is not identifiable; logit-sensitivity and logit-specificity were pooled separately using a DerSimonian–Laird random-effects model. These rows are exploratory. ᵇ All three studies rated at low overall risk of bias in the whole review address HF/LVSD, so this row is numerically identical to the HF/LVSD low-risk row above. CI = confidence interval; HF/LVSD = heart failure/left ventricular systolic dysfunction; HSROC = hierarchical summary receiver operating characteristic; NE = not estimable.

**Table 6:** Summary of findings (GRADE adapted for diagnostic test accuracy).

Outcome/Population	Studies (Patients)	Pooled Estimate (95% CI)	Implications per 1000 (Prevalence 10%)	Certainty (GRADE)	Reasons
HF/LVSD—sensitivity (primary)	5 (101,875)	0.86 (0.80–0.90)	86 of 100 true positives detected	Moderate	Downgraded for risk of bias (reference standard); robust to exclusion of high-risk studies
HF/LVSD—specificity (primary)	5 (101,875)	0.79 (0.70–0.85)	711 of 900 true negatives correctly classified	Moderate	Downgraded for risk of bias (reference standard); robust to exclusion of high-risk studies
All conditions—sensitivity (secondary)	12 (125,568)	0.84 (0.77–0.89)	84 of 100 true positives detected	Low	Downgraded for indirectness, inconsistency, and unexplained small-study effects
All conditions—specificity (secondary)	12 (125,568)	0.87 (0.79–0.93)	783 of 900 true negatives correctly classified	Low	Downgraded for indirectness, inconsistency, and unexplained small-study effects
Atrial fibrillation—sensitivity	2 (614)	Median 0.96 (0.91–1.00)	High accuracy, single condition	Very low (descriptive)	k < 4; downgraded for imprecision and indirectness
Atrial fibrillation—specificity	2 (614)	Median 0.94 (0.93–0.95)	High accuracy, single condition	Very low (descriptive)	k < 4; downgraded for imprecision and indirectness

Certainty was assessed with the GRADE framework adapted to diagnostic tests. The HF/LVSD subgroup is the pre-specified primary clinical question; the cross-condition estimate spans heterogeneous conditions and is descriptive. GRADE = Grading of Recommendations Assessment, Development, and Evaluation. The ‘Implications’ column is a heuristic illustration assuming a hypothetical disease prevalence of 10%; absolute numbers will vary with the true prevalence in the deployment setting.

## Data Availability

All datasets analysed during the current study are derived from the included publications. Extraction sheets and analytic code are available from the corresponding author upon reasonable request.

## Supplementary Materials

The following supporting information can be downloaded at: https://www.mdpi.com/article/10.3390/diagnostics16183014/s1: Table S1: PRISMA-DTA 2018 checklist (location of items in the manuscript); File S1: Database-specific search strategies (PubMed, Scopus, Web of Science, Embase); Table S2: Per-study 2 × 2 cell sourcing and data reconstruction notes; Table S3: Multiple-cohort or multiple-threshold studies: primary row selection; File S2: Pre-specified protocol deviations and rationale; Table S4: QUADAS-2 applicability concerns by domain (signalling-question summary); Table S5: Studies excluded at the full-text stage, with reasons; Table S6: Sensitivity analyses for the HF/LVSD subgroup; File S3: Statistical methods and R analytic pipeline (overview); File S4: PRISMA 2020 checklist.
