# Supplementary material for: Diagnostic Accuracy of Artificial Intelligence-Based Electrocardiography for the Detection of Heart Diseases: A Systematic Review and Meta-Analysis
Source: Diagnostics (Basel). 2026 Sep 17;16(18):3014. doi: 10.3390/diagnostics16183014 (PMC13605255; doi:10.3390/diagnostics16183014)
Supplement: Supplementary file 1 [file diagnostics-16-03014-s001.zip › diagnostics-4525524-supplementary.pdf]

Supplementary Material

Diagnostic accuracy of artificial intelligence-based electrocardiography for the detection of heart diseases: a systematic review and meta-analysis

Corresponding author: Joshuan J. Barboza (bmecajos@uss.edu.pe)

Contents

- Supplementary Table S1. PRISMA-DTA 2018 checklist (location of items in the manuscript).
- Supplementary File S1. Database-specific search strategies (PubMed, Scopus, Web of Science, Embase).
- Supplementary Table S2. Per-study 2×2 cell sourcing and data reconstruction notes.
- Supplementary Table S3. Multiple-cohort or multiple-threshold studies: primary row selection.
- Supplementary File S2. Pre-specified protocol deviations and rationale.
- Supplementary Table S4. QUADAS-2 applicability concerns by domain (signalling-question summary).
- Supplementary Table S5. Studies excluded at the full-text stage, with reasons.
- Supplementary Table S6. Sensitivity analyses for the HF/LVSD subgroup.
- Supplementary File S3. Statistical methods and R analytic pipeline (overview).
- Supplementary File S4. PRISMA 2020 checklist.

Supplementary Table S1. PRISMA-DTA checklist (selected items)

Where reported in the manuscript / supplementary file.

Section/topic	Item	Location
Title	1. Identify as DTA systematic review and meta-analysis.	Title
Abstract	2. Structured abstract (background, methods, results, conclusions).	Abstract
Introduction	3. Rationale, including knowledge gap.	Introduction
Introduction	4. Objectives with index test and target condition.	Introduction
Methods	5. Protocol and registration.	Methods—Protocol and registration
Methods	6. Eligibility criteria (PIRD).	Methods—Eligibility criteria
Methods	7. Information sources and search strategy.	Methods—Information sources; Supplementary File S1
Methods	8. Study selection (two reviewers, conflict resolution).	Methods—Study selection
Methods	9. Data extraction and items.	Methods—Data extraction; Supplementary Table S3
Methods	10. Risk of bias and applicability (QUADAS-2).	Methods—Risk of bias; Supplementary Table S6
Methods	11. Statistical analysis (bivariate model, HSROC).	Methods—Statistical analysis; Supplementary File S3
Methods	12. Publication bias (Deeks).	Methods—Statistical analysis; Figure 7
Methods	13. Certainty of evidence (GRADE adapted).	Methods—Statistical analysis; Table 5
Results	14. Study selection (PRISMA-DTA flow).	Results—Study selection; Figure 1
Results	15. Study characteristics.	Results; Table 1
Results	16. Risk of bias.	Results—Risk of bias; Figures 8–9; Table 4
Results	17. Per-study and pooled diagnostic accuracy.	Results; Tables 2–3; Figures 2–6
Results	18. Publication bias and small-study effects.	Results—Publication bias; Figure 7
Results	19. Sensitivity analyses and certainty.	Results; Table 5; Supplementary Table S8
Discussion	20. Interpretation, limitations, implications.	Discussion
Funding	21. Funding sources, declarations.	Declarations

Supplementary File S1. Database-specific search strategies

All searches were combined with the Boolean operator AND between concept blocks (Concept 1: artificial intelligence / deep learning; Concept 2: electrocardiography; Concept 3: heart diseases / target conditions). Limits: humans; English. Final search executed on 31 March 2026.

PubMed (n=130)

Concept 1: ("artificial intelligence"[MeSH] OR "deep learning"[MeSH] OR "machine learning"[MeSH] OR "neural networks, computer"[MeSH] OR "artificial intelligence"[tiab] OR "deep learning"[tiab] OR "machine learning"[tiab] OR "neural network*"[tiab] OR "convolutional"[tiab] OR "AI-ECG"[tiab] OR "ECG-AI"[tiab]).

Concept 2: ("electrocardiography"[MeSH] OR "electrocardiogram*"[tiab] OR "ECG"[tiab] OR "EKG"[tiab] OR "12-lead"[tiab] OR "single-lead"[tiab] OR "wearable"[tiab]).

Concept 3: ("heart diseases"[MeSH] OR "cardiovascular diseases"[MeSH] OR "heart failure"[MeSH] OR "ventricular dysfunction, left"[MeSH] OR "atrial fibrillation"[MeSH] OR "coronary artery disease"[MeSH] OR "myocardial infarction"[MeSH] OR "cardiomyopathy, hypertrophic"[MeSH] OR "hypertrophy, left ventricular"[MeSH] OR "pulmonary embolism"[MeSH] OR "heart failure"[tiab] OR "low ejection fraction"[tiab] OR "left ventricular systolic dysfunction"[tiab] OR "atrial fibrillation"[tiab] OR "coronary artery disease"[tiab] OR "STEMI"[tiab] OR "NSTEMI"[tiab] OR "hypertrophic cardiomyopathy"[tiab] OR "pulmonary embolism"[tiab]).

Filters: Humans; English. Final search: 31 March 2026.

Scopus

TITLE-ABS-KEY((("artificial intelligence" OR "deep learning" OR "machine learning" OR "neural network*" OR "convolutional" OR "AI-ECG" OR "ECG-AI")) AND (("electrocardiogram*" OR "ECG" OR "12-lead" OR "single-lead" OR "wearable")) AND (("heart failure" OR "left ventricular dysfunction" OR "low ejection fraction" OR "atrial fibrillation" OR "coronary artery disease" OR "myocardial infarction" OR "STEMI" OR "hypertrophic cardiomyopathy" OR "left ventricular hypertrophy" OR "pulmonary embolism" OR "heart disease*"))) AND PUBYEAR > 2014.

Web of Science (Core Collection)

TS=("artificial intelligence" OR "deep learning" OR "machine learning" OR "neural network*" OR "AI-ECG") AND TS=("electrocardio*" OR "ECG") AND TS=("heart failure" OR "ejection fraction" OR "atrial fibrillation" OR "coronary artery disease" OR "myocardial infarction" OR "hypertrophic cardiomyopathy" OR "left ventricular hypertrophy" OR "pulmonary embolism").

Embase

(('artificial intelligence'/exp OR 'deep learning'/exp OR 'machine learning'/exp OR 'neural network'/exp) AND ('electrocardiography'/exp OR ecg:ab,ti) AND ('heart failure'/exp OR 'atrial fibrillation'/exp OR 'coronary artery disease'/exp OR 'myocardial infarction'/exp OR 'hypertrophic cardiomyopathy'/exp OR 'left ventricular hypertrophy'/exp OR 'pulmonary embolism'/exp)) AND [english]/lim AND [humans]/lim.

Supplementary Table S2. Per-study 2×2 cell sourcing and reconstruction notes

Study	Cell source	Notes
Adedinsewo 2024	Reported	Confusion matrix in Figure 3B of the original article; LVEF<50% endpoint used as primary.
Carter 2026	Reported	Confusion matrix in Figure 2A; cells confirmed against PRISMA-style flow (Figure 1).
König 2024	Reported (Fig. 2 fractions)	Cells derived from study-reported sensitivity, specificity and total N at the optimal Youden threshold.
Lee 2025	Reconstructed (approximate)	Reconstructed from reported sensitivity, specificity and patient denominators (Korean and US cohorts).
Thambiraj 2026	Reported	Cells from binary classification at LVEF≤40%; ECG-only model selected for primary synthesis.
Fiorina 2024	Reconstructed (approximate)	Reconstructed from per-arm sensitivity/specificity, with N reflecting evaluable smartwatch ECGs.
Lueken 2025	Reconstructed	Reconstructed from PPV/NPV and prevalence at the operating point of the deployed CNN.
Tang 2023	Reported	8-lead model used for primary row (single-lead variants excluded to avoid double counting).
Luo 2026 (STEMI)	Reported	STEMI cohort used as primary; NSTEMI/UA/AD as exploratory (Supplementary Table S4).
Siontis 2023	Reconstructed	Reconstructed from PPV/NPV and prevalence in derivation cohort (case–control).
Huang 2025	Reconstructed (approximate)	Reconstructed from CatBoost reported sensitivity, specificity and test-set N.
Valente Silva 2023	Reconstructed (exact)	Specificity=1.00 ⇒ FP=0; consistent with reported PPV/NPV.
Karabayir 2024	Reported	Excluded from primary synthesis (case–control, ICD reference).
Awasthi 2023	Reconstructed (approximate)	Excluded from primary synthesis (case–control, artefactual prevalence).
Sun 2021	Not derivable	Only Se/Sp/AUC reported without denominators; excluded from quantitative synthesis.
Park 2023	No denominator	Cells not derivable from reported metrics; excluded from quantitative synthesis.
Chang 2021	No specificity	Specificity per class not reported in main text; excluded from quantitative synthesis.
Babur Guler 2026	No controls	Case-only cohort; cells not estimable.

Liu 2023	Reported	Multi-label model; LVH class extracted but excluded from primary synthesis (expert-ECG reference, low prevalence).
Díaz-Herrera 2025	Reconstructed (approximate)	Excluded from primary synthesis (small sample, derivation, ACS spectrum mismatch).

Supplementary Table S3. Studies with multiple cohorts, thresholds, or models: primary row selected

Study	Decision	Rationale
Adedinsewo 2024	Primary: LVEF<50%	Pre-specified primary endpoint of the original article; LVEF<45% retained as secondary.
Luo 2026	Primary: STEMI cohort	Representative ACS phenotype with discrete electrocardiographic signature; NSTEMI/UA/AD are exploratory and share controls.
Karabayir 2024	Excluded	Case–control, ICD-coded reference; double-counts UTHSC and AHWFB cohorts of the same model.
Thambiraj 2026	Primary: ECG−only 2D−CNN at LVEF≤40%	Avoids overlap with hybrid model trained on the same patient set.
Tang 2023	Primary: 8-lead SE-ResNet-50	Avoids overlap with the single-lead model on the same patients.
König 2024 / Lee 2025	Primary threshold: LVEF<40%	Other thresholds report only AUC and are not directly comparable.

Supplementary File S2. Pre-specified protocol deviations and rationale

This file documents deviations from the registered protocol identified during the synthesis phase and provides their rationale. Decisions follow the Cochrane Handbook for DTA reviews and the PRISMA-DTA statement.

Type of meta-analysis. The protocol describes a diagnostic test accuracy (DTA) review. We therefore used the bivariate random-effects (Reitsma) model on 2×2 cells, with HSROC visualisation; no intervention-style metabin/metacont models were used.

Scope across conditions. Although the registered protocol was framed broadly as 'heart diseases', target conditions varied across studies. We pre-specified a primary subgroup for HF/LVSD (k=5) and presented descriptive synthesis for other conditions where k<4.

Reference standard heterogeneity. Studies using expert ECG adjudication as the reference (Chang 2021, Liu 2023, in part Fiorina 2024) are conceptually closer to inter-reader agreement than to anatomical truth. Where this was the only reference, we documented the concern and either retained the study with a flag (atrial fibrillation) or excluded it from quantitative synthesis (Chang 2021, Liu 2023, Babur Guler 2026).

Reconstruction of 2×2 cells. Cells were reconstructed when needed and the source is reported in Supplementary Table S3.

Unit-of-analysis decisions. When more than one cohort, threshold, or model was reported per study, one primary row was pre-specified (Supplementary Table S4).

Diagnostic case–control designs. Awasthi 2023, Karabayir 2024, and Díaz-Herrera 2025 were excluded from the primary synthesis because of artefactual prevalence; their estimates are presented descriptively only.

Publication bias test. Deeks test was performed for the primary synthesis (k=12); subgroup-level Deeks was not performed because each subgroup had k<10.

GRADE. GRADE for diagnostic tests was applied post hoc (not specified in the original protocol). Domains downgraded are recorded in Table 5.

Search update. The final search was conducted on 31 March 2026. Eligible publications appearing after this date may not be included; the protocol allows for an update.

Supplementary Table S4. QUADAS-2 applicability concerns by domain

Study	Applicability D1 (patient selection)	Applicability D2 (index test)	Applicability D3 (reference standard)
Adedinsewo 2024	Low (obstetric population may limit generalisability)	Low	Low
Awasthi 2023	High (case–control, artefactual prevalence)	Low	Unclear
Babur Guler 2026	High (case-only, no controls)	Unclear	High
Carter 2026	Low (4 geographically diverse sites)	Low	Low
Chang 2021	High (curated multi-class dataset)	Low	High (expert ECG reference)
Díaz-Herrera 2025	High (small derivation cohort, ACS only)	High	Low
Fiorina 2024	Unclear (single-centre)	Low	Unclear (expert ECG)
Huang 2025	Low	Unclear (engineered features)	Unclear
Karabayir 2024	High (case–control)	Unclear	High (ICD reference)
König 2024	Low	Low	Low
Lee 2025	Low (external MIMIC-IV)	High (image-based pipeline)	High (discharge-note LVEF)
Liu 2023	Low	Unclear (image input)	High (expert ECG)
Lueken 2025	Unclear (handheld device cohort)	Unclear	High (expert single-lead)
Luo 2026	Unclear	Unclear	Low
Park 2023	Unclear (stable angina only)	High	Low
Siontis 2023	High (case–control derivation)	Unclear	Low
Sun 2021	Low	Unclear	Low
Tang 2023	Unclear	High (non-standard 8-lead)	Low
Thambiraj 2026	Unclear	High (NLP-extracted reference)	High
Valente Silva 2023	Low	Unclear	Low

Supplementary Table S5. Studies excluded at the full-text stage, with reasons (n=24)

Reason	Count	Notes
Conference abstract / no full text	16	Posters and meeting abstracts without a peer-reviewed full text.
Holter / ambulatory recording analysis only	2	Index test scope outside resting 12-lead / single-lead ECG.
Non-standard lead set / 8-lead + liver transplant population	2	Ineligible population or non-comparable input.
Signal-only / segment-level analysis	1	No patient-level diagnostic outcome.
P-wave-only analysis	1	Restricted to a single ECG feature, not patient-level diagnosis.
Predictive (non-diagnostic) model	1	Prognostic prediction without a target condition diagnosis.
Single-lead-only design ineligible for 2×2	1	Insufficient information to reconstruct 2×2.

Supplementary Table S6. Sensitivity analyses for the HF/LVSD subgroup

Sensitivity analysis	k	Sensitivity (95% CI)	Specificity (95% CI)	AUC
Primary subgroup (all 5 studies)	5	0.86 (0.80–0.90)	0.79 (0.70–0.85)	0.89
Excluding small prospective study (Adedinsewo 2024)	4	0.86 (0.78–0.92)	0.77 (0.69–0.84)	0.88
Excluding studies with reference adjudication concerns (Lee 2025, Thambiraj 2026)	3	0.84 (0.74–0.91)	0.81 (0.71–0.88)	0.90
Restricting to LVEF≤40% threshold	3	0.86 (0.80–0.91)	0.77 (0.66–0.85)	0.88

Supplementary File S3. Statistical methods and R analytic pipeline (overview)

All analyses were performed in R version 4.3. Per-study sensitivity and specificity were computed from 2×2 cells with Clopper–Pearson 95% confidence intervals. The bivariate random-effects model (Reitsma) jointly modelled sensitivity and specificity on the logit scale using the package mada, and the corresponding HSROC curve was derived following the Rutter–Gatsonis parameterisation. Pooled estimates with 95% confidence intervals are reported for sensitivity, specificity, and the HSROC area under the curve. The Deeks test was implemented as the regression of ln(diagnostic odds ratio) on the inverse square-root of the effective sample size (ESS), weighted by ESS, with $p<0.10$ considered indicative of asymmetry.

Per-study and subgroup forest plots paired sensitivity and specificity; HSROC plots presented study points sized by the effective sample size with a fitted summary curve. Subgroups were defined a priori by target condition; bivariate pooling was conducted only when $k \geq 4$ within a subgroup. Sensitivity analyses excluded studies at high risk of bias or with case-control sampling. R scripts (00_setup.R; 01_prisma.R; 02_dta_overall.R; 03_dta_subgroups.R; 04_sesgo_publicacion.R; 05_quadas2.R; 06_tablas_caracteristicas.R; lib_forest.R; run_all.R) are available from the corresponding author on reasonable request.

Reporting was aligned with PRISMA-DTA (Supplementary Table S1). Risk of bias and applicability concerns were assessed in duplicate using the QUADAS-2 tool (Supplementary Table S6). Certainty of evidence was rated with the GRADE framework adapted to diagnostic tests (Table 5). The protocol followed the principles of PROSPERO prospective registration.

Supplementary File S4. PRISMA 2020 checklist.

Completed PRISMA 2020 main checklist and PRISMA 2020 for Abstracts checklist. Locations refer to the manuscript and accompanying tables/supplementary files.

PRISMA 2020 Main Checklist

Topic	No.	Item	Location where item is reported
**TITLE**			
**Title**	1	Identify the report as a systematic review.	1
**ABSTRACT**			
**Abstract**	2	See the PRISMA 2020 for Abstracts checklist	1-2
**INTRODUCTION**			
**Rationale**	3	Describe the rationale for the review in the context of existing knowledge.	3
**Objectives**	4	Provide an explicit statement of the objective(s) or question(s) the review addresses.	3
**METHODS**			
**Eligibility criteria**	5	Specify the inclusion and exclusion criteria for the review and how studies were grouped for the syntheses.	4
**Information sources**	6	Specify all databases, registers, websites, organisations, reference lists and other sources searched or consulted to identify studies. Specify the date when each source was last searched or consulted.	Pages 4–5; Supplementary File S1, Supplementary Material, page 3

Topic	No.	Item	Location where item is reported
**Search strategy**	7	Present the full search strategies for all databases, registers and websites, including any filters and limits used.	Page 5; Supplementary File S1, Supplementary Material, pages 3–4
**Selection process**	8	Specify the methods used to decide whether a study met the inclusion criteria of the review, including how many reviewers screened each record and each report retrieved, whether they worked independently, and if applicable, details of automation tools used in the process.	5
**Data collection process**	9	Specify the methods used to collect data from reports, including how many reviewers collected data from each report, whether they worked independently, any processes for obtaining or confirming data from study investigators, and if applicable, details of automation tools used in the process.	5
**Data items**	10a	List and define all outcomes for which data were sought. Specify whether all results that were compatible with each outcome domain in each study were sought (e.g. for all measures, time points, analyses), and if not, the methods used to decide which results to collect.	Pages 5–6; Table 2, Tables file, page 3
	10b	List and define all other variables for which data were sought (e.g. participant and intervention characteristics, funding sources). Describe any assumptions made about any missing or unclear information.	Page 5; Table 1, Tables file, pages 1–2; Supplementary Table S3, Supplementary Material, pages 5–6
**Study risk of bias assessment**	11	Specify the methods used to assess risk of bias in the included studies, including details of the tool(s) used, how many reviewers assessed each study and whether they worked independently, and if applicable, details of automation tools used in the process.	Page 5; Table 4, Tables file, page 5; Supplementary Table S6, Supplementary Material, page 9

Topic	No.	Item	Location where item is reported
**Effect measures**	12	Specify for each outcome the effect measure(s) (e.g. risk ratio, mean difference) used in the synthesis or presentation of results.	Page 6; Table 2, Tables file, page 3; Table 3, Tables file, page 4
**Synthesis methods**	13a	Describe the processes used to decide which studies were eligible for each synthesis (e.g. tabulating the study intervention characteristics and comparing against the planned groups for each synthesis (item 5)).	Pages 5–6; Supplementary Table S4, Supplementary Material, page 7
	13b	Describe any methods required to prepare the data for presentation or synthesis, such as handling of missing summary statistics, or data conversions.	Page 5; Supplementary Table S3, Supplementary Material, pages 5–6
	13c	Describe any methods used to tabulate or visually display results of individual studies and syntheses.	Page 6; Tables 1–3, Tables file, pages 1–4; Figures 2–6
	13d	Describe any methods used to synthesize results and provide a rationale for the choice(s). If meta-analysis was performed, describe the model(s), method(s) to identify the presence and extent of statistical heterogeneity, and software package(s) used.	Page 6; Supplementary File S3, Supplementary Material, page 12
	13e	Describe any methods used to explore possible causes of heterogeneity among study results (e.g. subgroup analysis, meta-regression).	Page 6; Results pages 8–9; Figures 3 and 5
	13f	Describe any sensitivity analyses conducted to assess robustness of the synthesized results.	Page 6; Results page 9; Supplementary Table S8, Supplementary Material, page 11
**Reporting bias assessment**	14	Describe any methods used to assess risk of bias due to missing results in a synthesis (arising from reporting biases).	Page 6; Results page 9; Figure 7

Topic	No.	Item	Location where item is reported
**Certainty assessment**	15	Describe any methods used to assess certainty (or confidence) in the body of evidence for an outcome.	Page 6; Results page 9; Table 5, Tables file, page 6
**RESULTS**			
**Study selection**	16a	Describe the results of the search and selection process, from the number of records identified in the search to the number of studies included in the review, ideally using a flow diagram.	Page 6; Figure 1
	16b	Cite studies that might appear to meet the inclusion criteria, but which were excluded, and explain why they were excluded.	Page 6; Figure 1; Supplementary Table S7, Supplementary Material, page 10
**Study characteristics**	17	Cite each included study and present its characteristics.	Pages 6–7; Table 1, Tables file, pages 1–2
**Risk of bias in studies**	18	Present assessments of risk of bias for each included study.	Page 7; Table 4, Tables file, page 5; Figures 8–9
**Results of individual studies**	19	For all outcomes, present, for each study: (a) summary statistics for each group (where appropriate) and (b) an effect estimate and its precision (e.g. confidence/credible interval), ideally using structured tables or plots.	Pages 7–9; Table 2, Tables file, page 3; Figures 2, 4, and 6
**Results of syntheses**	20a	For each synthesis, briefly summarise the characteristics and risk of bias among contributing studies.	Pages 7–8
	20b	Present results of all statistical syntheses conducted. If meta-analysis was done, present for each the summary estimate and its precision (e.g. confidence/credible interval) and measures of statistical heterogeneity. If comparing groups, describe the direction of the effect.	Pages 7–9; Table 3, Tables file, page 4; Figures 2–5

Topic	No.	Item	Location where item is reported
	20c	Present results of all investigations of possible causes of heterogeneity among study results.	Pages 8–10; Figures 3 and 5
	20d	Present results of all sensitivity analyses conducted to assess the robustness of the synthesized results.	Page 9; Supplementary Table S8, Supplementary Material, page 11
**Reporting biases**	21	Present assessments of risk of bias due to missing results (arising from reporting biases) for each synthesis assessed.	9
**Certainty of evidence**	22	Present assessments of certainty (or confidence) in the body of evidence for each outcome assessed.	Page 9; Table 5, Tables file, page 6
**DISCUSSION**			
**Discussion**	23a	Provide a general interpretation of the results in the context of other evidence.	Pages 9–10
	23b	Discuss any limitations of the evidence included in the review.	Pages 10–11
	23c	Discuss any limitations of the review processes used.	Page 11
	23d	Discuss implications of the results for practice, policy, and future research.	Page 11
**OTHER INFORMATION**			
**Registration and protocol**	24a	Provide registration information for the review, including register name and registration number, or state that the review was not registered.	Page 2; Page 4
	24b	Indicate where the review protocol can be accessed, or state that a protocol was not prepared.	4

Topic	No.	Item	Location where item is reported
	24c	Describe and explain any amendments to information provided at registration or in the protocol.	Page 5; Supplementary File S2, Supplementary Material, page 8
**Support**	25	Describe sources of financial or non-financial support for the review, and the role of the funders or sponsors in the review.	12
**Competing interests**	26	Declare any competing interests of review authors.	12
**Availability of data, code and other materials**	27	Report which of the following are publicly available and where they can be found: template data collection forms; data extracted from included studies; data used for all analyses; analytic code; any other materials used in the review.	Page 12; Supplementary File S3, Supplementary Material, page 12

PRIMSA Abstract Checklist

Topic	No.	Item	Reported?
**TITLE**			
**Title**	1	Identify the report as a systematic review.	Yes
**BACKGROUND**			
**Objectives**	2	Provide an explicit statement of the main objective(s) or question(s) the review addresses.	Yes
**METHODS**			
**Eligibility criteria**	3	Specify the inclusion and exclusion criteria for the review.	Yes
**Information sources**	4	Specify the information sources (e.g. databases, registers) used to identify studies and the date when each was last searched.	Yes
**Risk of bias**	5	Specify the methods used to assess risk of bias in the included studies.	Yes
**Synthesis of results**	6	Specify the methods used to present and synthesize results.	Yes
**RESULTS**			
**Included studies**	7	Give the total number of included studies and participants and summarise relevant characteristics of studies.	Yes
**Synthesis of results**	8	Present results for main outcomes, preferably indicating the number of included studies and participants for each. If meta-analysis was done, report the summary estimate and confidence/credible interval. If comparing groups, indicate the direction of the effect (i.e. which group is favoured).	Yes
**DISCUSSION**			

Topic	No.	Item	Reported?
**Limitations of evidence**	9	Provide a brief summary of the limitations of the evidence included in the review (e.g. study risk of bias, inconsistency and imprecision).	Yes
**Interpretation**	10	Provide a general interpretation of the results and important implications.	Yes
**OTHER**			
**Funding**	11	Specify the primary source of funding for the review.	Yes
**Registration**	12	Provide the register name and registration number.	Yes

From: Page MJ, McKenzie JE, Bossuyt PM, Boutron I, Hoffmann TC, Mulrow CD, et al. The PRISMA 2020 statement: an updated guideline for reporting systematic reviews. MetaArXiv. 2020, September 14. DOI: 10.31222/osf.io/v7gm2. For more information, visit: www.prisma-statement.org
